# A comprehensive evaluation of ensembl, RefSeq, and UCSC annotations in the context of RNA-seq read mapping and gene quantification

**DOI:** 10.1186/s12864-015-1308-8

**Published:** 2015-02-18

**Authors:** Shanrong Zhao, Baohong Zhang

**Affiliations:** Clinical Genetics and Bioinformatics, BioTherapeutics Clinical R&D, Pfizer Worldwide Research & Development, Cambridge, MA 02139 USA

**Keywords:** RNA-Seq, Gene quantification, Gene model, RefSeq, UCSC, Ensembl

## Abstract

**Background:**

RNA-Seq has become increasingly popular in transcriptome profiling. One aspect of transcriptome research is to quantify the expression levels of genomic elements, such as genes, their transcripts and exons. Acquiring a transcriptome expression profile requires genomic elements to be defined in the context of the genome. Multiple human genome annotation databases exist, including RefGene (RefSeq Gene), Ensembl, and the UCSC annotation database. The impact of the choice of an annotation on estimating gene expression remains insufficiently investigated.

**Results:**

In this paper, we systematically characterized the impact of genome annotation choice on read mapping and transcriptome quantification by analyzing a RNA-Seq dataset generated by the Human Body Map 2.0 Project. The impact of a gene model on mapping of non-junction reads is different from junction reads. For the RNA-Seq dataset with a read length of 75 bp, on average, 95% of non-junction reads were mapped to exactly the same genomic location regardless of which gene models was used. By contrast, this percentage dropped to 53% for junction reads. In addition, about 30% of junction reads failed to align without the assistance of a gene model, while 10–15% mapped alternatively. There are 21,958 common genes among RefGene, Ensembl, and UCSC annotations. When we compared the gene quantification results in RefGene and Ensembl annotations, 20% of genes are not expressed, and thus have a zero count in both annotations. Surprisingly, identical gene quantification results were obtained for only 16.3% (about one sixth) of genes. Approximately 28.1% of genes’ expression levels differed by 5% or higher, and of those, the relative expression levels for 9.3% of genes (equivalent to 2038) differed by 50% or greater. The case studies revealed that the gene definition differences in gene models frequently result in inconsistency in gene quantification.

**Conclusions:**

We demonstrated that the choice of a gene model has a dramatic effect on both gene quantification and differential analysis. Our research will help RNA-Seq data analysts to make an informed choice of gene model in practical RNA-Seq data analysis.

**Electronic supplementary material:**

The online version of this article (doi:10.1186/s12864-015-1308-8) contains supplementary material, which is available to authorized users.

## Background

RNA-Seq, the sequencing of a population of RNA transcripts using high-throughput sequencing technologies, profiles an entire transcriptome at single-base resolution whilst concurrently quantifying gene expression levels [1-5]. RNA-Seq can analyze subtle features of the transcriptome, such as novel transcript variants, allele-specific expression, and splice junctions [4,5]. Previously, we performed a side-by-side comparison of RNA-Seq and microarray to investigate T-cell activation, and demonstrated that RNA-Seq is superior in detecting low abundance transcripts, and for differentiating biologically critical isoforms [6]. RNA-Seq also avoids technical limitations inherent to the microarray platform related to probe performance, such as cross-hybridization, limited detection range of individual probes, as well as non-specific hybridization [6-8]. With decreasing sequencing cost, RNA-Seq is becoming an attractive approach to profile gene expression or transcript abundance, and to evaluate differential expression among biological conditions.

Current RNA-Seq approaches use shotgun sequencing technologies such as Illumina, in which millions or even billions of short reads are generated from a randomly fragmented cDNA library. After sequencing, the first step involves mapping those short reads to a genome or transcriptome. In recent years, a large number of mapping algorithms have been developed for read mapping and RNA-Seq differential analysis [9-14]. However, accurate alignment of high-throughput short RNA-Seq reads remains challenging, mainly because of junction (i.e., exon-exon spanning) reads and the ambiguity of multiple-mapping reads. Currently, many RNA-Seq alignment tools, including GSNAP [15], OSA [16], STAR [17], MapSplice[18], and TopHat [19], use reference transcriptomes to inform the alignments of junction reads. In our previous study [20], we had assessed the impact of using RefGene (RefSeq Gene) [21] on mapping short RNA-Seq reads, and demonstrated that without the assistance of RefGene, more than one third of junction reads failed to map to the reference genome in the alignment process.

One aspect of transcriptome research is to quantify expression levels of genes, transcripts, and exons. Acquiring the transcriptome expression profile requires genomic elements to be defined in the context of the genome. In addition to RefGene, there are several other public human genome annotations, including UCSC Known Genes [22], Ensembl [23], AceView [24], Vega [25], and GENCODE[26]. Characteristics of these annotations differ because of variations in annotation strategies and information sources. RefSeq human gene models are well supported and broadly used in various studies. The UCSC Known Genes dataset is based on protein data from Swiss-Prot/TrEMBL (UniProt) and the associated mRNA data from GenBank, and serves as a foundation for the UCSC Genome Browser. Vega genes are manually curated transcripts produced by the HAVANA group at the Welcome Trust Sanger Institute, and are merged into Ensembl. Ensembl genes contain both automated genome annotation and manual curation, while the gene set of GENCODE corresponds to Ensembl annotation since GENCODE version 3c (equivalent to Ensembl 56). AceView provides a comprehensive non-redundant curated representation of all available human cDNA sequences.

Although there are multiple genome annotations available, researchers need to choose a genome annotation (or gene model) while performing RNA-Seq data analysis. However, the effect of genome annotation choice on downstream RNA-Seq expression estimates is under-appreciated. Wu *et al*. [27] defined the complexity of human genome annotations in terms of the number of genes, isoforms, and exons, and demonstrated that the selection of human genome annotation results in different gene expression estimates. Chen *et al*. [28] systematically compared the human annotations present in RefSeq, Ensembl, and AceView on diverse transcriptomic and genetic analyses. They found that the human gene annotations in the three databases are far from complete, although Ensembl and AceView annotate many more genes than RefSeq. In this paper, we performed a comprehensive evaluation of different annotations on RNA-Seq data analysis, including RefGene, UCSC, and Ensembl. We chose these three gene models because we use them regularly for in-house RNA-Seq data analysis. Our research focused on: (1) comparing the coverage and incompleteness of different gene models; (2) quantifying the impact of gene models on the mapping of both junction and non-junction reads; and (3) evaluating the effect of genome annotation choice on gene quantification and differential analysis. To a broader extent, one of the most practical questions researchers want to know in advance is: if different gene models are chosen for RNA-Seq data analysis, what is the chance of obtaining the same quantification result for a given gene?

## Results and discussion

The Human Body Map 2.0 Project generated RNA-Seq data for 16 different human tissues (adipose, adrenal, brain, breast, colon, heart, kidney, leukocyte, liver, lung, lymph node, ovary, prostate, skeletal muscle, testis, and thyroid). We chose to analyze this public dataset because gene expression is tissue specific and analyzing those 16 high-quality RNA-Seq samples as a whole could result in less biased conclusions. Note that none of the gene annotation is 100% complete. As a result, for those RNA-Seq reads not covered by a gene annotation, whether to use the gene model in the mapping step has no impact on their mappings. Therefore, to fairly assess the impact of a gene model on RNA-Seq read mapping, only those reads covered by a gene model were used. In this study, we devised a two-stage mapping protocol. In Stage #1, all reads that are not covered by a gene model were filtered out. In Stage #2, all remaining reads were mapped to the reference genome with and without the use of a gene model. The role of a gene model in the mapping step was then quantified and characterized by comparing the mapping results in Stage #2.

### The coverage of different gene annotations

**Figure 1:**
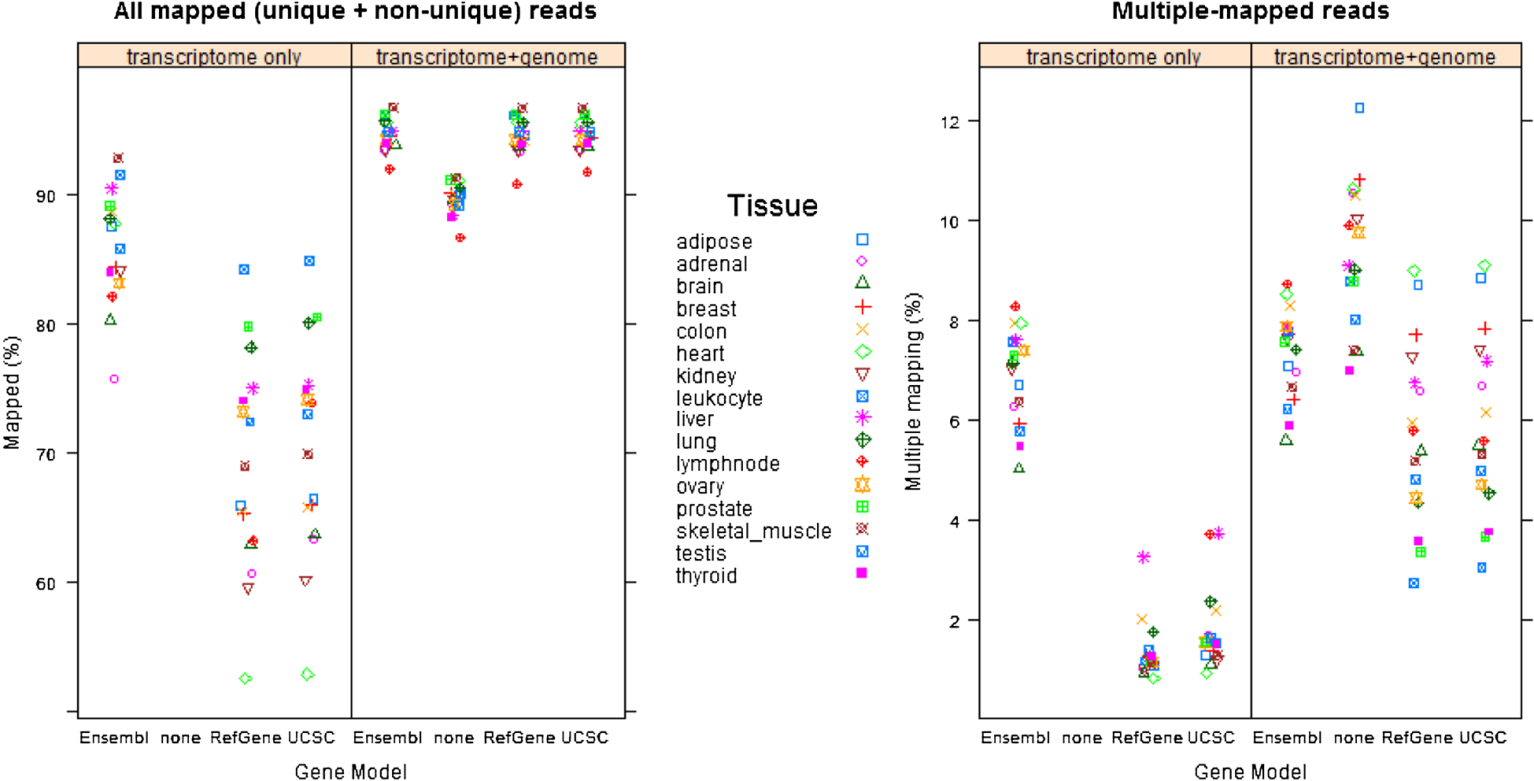
**The read mapping summary for 16 tissue samples in the** **“transcriptome only”** **and** **“transcriptome** **+** **genome”** **mapping modes (note**: **read length** **=** **75 bp).** In the “transcriptome only” mode, more reads are mapped in Ensembl than in RefGene and UCSC (left panel), and more reads become multiple-mapped in Ensembl than in RefGene and UCSC (right panel). Note: the gene model “none” means the RNA-Seq reads are mapped to the reference genome directly without the use of a gene model.

The RNA-Seq read mapping summaries for all 16 samples were shown in Additional file 1: Table S1 (read length = 75 bp) and Additional file 1: Table S2 (read length = 50 bp), respectively. There are two different mapping modes in Additional file 1: Tables S1 and S2. In the “transcriptome only” mapping mode, all RNA-Seq reads were mapped to a reference transcriptome only. If a read could not be mapped to a known gene region, it becomes unmapped, even though it could potentially be aligned to a genomic region without annotations. While in the “transcriptome + genome” mapping mode, reads were first mapped to a reference transcriptome, and then the unmapped ones were mapped to the reference genome. The impact of a reference transcriptome on the mapping of RNA-Seq reads is attenuated in the “transcriptome + genome” mapping mode because every unmapped read has a second chance to be mapped to a genome. The mapping summaries for the data in Additional file 1: Tables S1 and S2 were shown in Figure 1 and Additional file 1: Figure S1, respectively. In the “transcriptome only” mapping mode, more reads were mapped in Ensembl than in RefGene and/or UCSC. For each tissue type, the mapping rate was similar between RefGene and UCSC. The average read mapping rates were 86%, 69%, and 70% for Ensembl, RefGene, and UCSC annotations, respectively. Short-read mapping is a basic step in RNA-Seq data analyses, and to a certain extent, the percent of reads mapped to a given transcriptome can roughly reflect the completeness of its annotated genes and transcripts. Thus, Ensembl annotation has much broader gene coverage than RefGene and UCSC.

In contrast, Figure 1 shows that the read mapping percentage is also sample dependent, and this holds true for every gene model. For instance, only 52.5% of sequence reads in the heart were mapped to the RefGene model; while in leukocytes, 84.2% of reads could be mapped to RefGene. This mapping difference between heart and leukocyte results from, at least in part, the incompleteness of the RefGene annotation. As more genes are annotated in a gene model, a higher percentage of reads will be mapped in the “Transcriptome only” mapping mode.

The data patterns in “transcriptome + genome” mapping mode were different from those determined by the “transcriptome only” mode (left panel on Figure 1). In the “transcriptome + genome” mapping mode, the average mapping rates for Ensembl, RefGene, and UCSC increased to 96.7%, 94.5%, and 94.6%, respectively, and the mapping rate difference among different gene models decreased. This large difference in the mapping rates between the two modes suggests the incompleteness of gene models: there are many reads that were mapped to the genomic regions without annotations.

In the “transcriptome only” mapping mode, an average of 6.9%, 1.4%, and 1.8% of reads were multiple-mapped reads in Ensembl, RefGene, and UCSC gene models, respectively (the right panel in Figure 1). The percentage of multiple-mapped reads in Ensembl is higher than in RefGene or UCSC. Usually, a more comprehensive annotation generally annotates more genes and isoforms, and thus, increases the possibility of ambiguous mappings. These ambiguous mappings directly translate to an increase in the percentage of non-uniquely mapped reads.

### The impact of a gene model on RNA-seq read mapping

**Figure 2:**
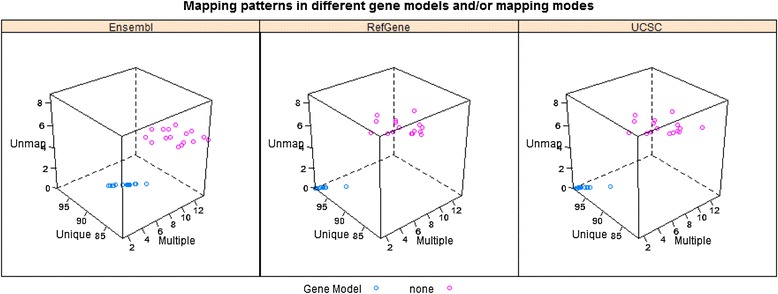
**The effect of a gene model on the mapping summaries for 16 tissue samples (read length** **=** **75 bp).** The RefGene and UCSC consistently have the highest percentage of uniquely mapped reads; while the percentage of non-uniquely mapped reads is much higher in Ensembl. Without a gene model (indicated in pink) in the mapping step, a constant 6% of reads become unmapped.

In Stage #1, the unmapped reads from the “transcriptome only” mapping mode were filtered out. In Stage #2, we remapped the remaining reads with and without the use of gene models. When gene models were used in Stage #2, all reads could be mapped, either uniquely or to multiple locations, and there were no unmapped reads. When those reads were remapped to genome without the use of gene models, some became unmapped. According to the number of mapped locations (#ML), all sequence reads were classified into three categories, unique (i.e., #ML = 1), multiple (i.e., #ML > =2), and unmapped (i.e., #ML = 0). The RNA-Seq reads remapping summaries in Stage #2 for all 16 samples were shown in Figure 2 (read length = 75 bp) and Additional file 1: Figure S2 (read length = 50 bp), respectively. The numeric data corresponding to Figure 2 and Additional file 1: Figure S2 were tabulated in Additional file 1: Tables S3 and S4, respectively. The RefGene and UCSC consistently had the highest percentage of uniquely mapped reads; while the percentage of non-uniquely mapped reads was much higher in Ensembl (samples colored in blue in Figure 2). Without a gene model, the percentage of unmapped reads was nearly constant at 6% (samples colored in pink in Figure 2). As we demonstrated as follows, a gene model mainly affects the alignment of junction reads, but has little impact on non-junction reads. On average, 23% of reads in our samples were junction reads, and usually about one third of them failed to be mapped without the use of a gene model. Therefore, it is expected that ~6% (23% * 0.33) of the mapped reads become unmapped without the use of a gene model.

**Figure 3:**
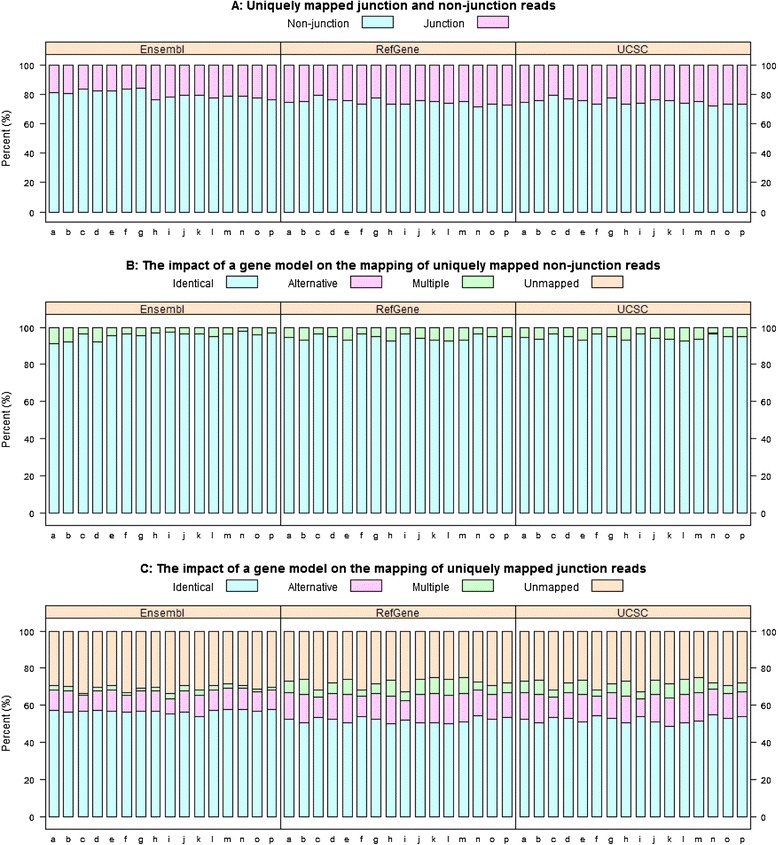
**The impact of a gene model on RNA-** **Seq read mapping (read length** **=** **75 bp). (A)** composition of mapped reads: roughly 23% are junction reads, and the rest 77% are non-junction reads; **(B)** effect on mapping of non-junctions reads: on average, 95% remain mapped to exactly the same genomic location, whilst 3–9% of reads become multiple-mapped reads; **(C)** effect on mapping of junctions reads: an average of 53% of reads remain mapped to the same genomic regions without the assistance of a gene model. About 30% of junction reads fail to be mapped, while 10–15% map alternatively. (Note: the 16 tissue sample names are denoted as follows: **a**: adipose; **b**: adrenal, **c**: brain; **d**: breast; **e**: colon; **f**: heart; **g**: kidney; **h**: leukocyte; **i**: liver; **j**: lung; **k**: lymph node; **l**: ovary; **m**: prostate; **n**: skeletal muscle; **o**: testis; and **p**: thyroid).

To evaluate the impact of a gene model on read mapping, the mapping summaries in Figure 2 and Additional file 1: Figure S2 were not sufficient. For instance, a read could be aligned differently with and without the assistance of a gene model in mapping, and in this scenario, the mapping summary could not identify such a difference. Thus, we compared the mapping details for every read, including start and end positions, and splicing sites. For simplicity, in Stage #2, we focused on uniquely mapped reads in the “transcriptome only” mapping mode. A uniquely mapped read could be classified into four categories according to its corresponding mapping information without a gene model: (1) “Identical”—remaining mapped to the same genomic region; (2) “Alternative”—still uniquely mapped but differently; (3) “Multiple”—mapped to more locations; and (4) “Unmapped”. The detailed evaluation results are summarized in Figure 3 (read length = 75 bp) and Additional file 1: Figure S3 (read length = 50 bp), and reported in Additional file 1: Tables S5 and S6.

In Figure 3A, we divided uniquely mapped reads into two classes, i.e., non-junction reads and junction reads, and investigated the impact of a gene model on their mapping. Accordingly to Figure 3A, roughly 23% of mapped reads were junction reads, and the remaining 77% were non-junction reads. For non-junction reads (see Figure 3B), 95% remained mapped to exactly the same genomic location regardless of the use of a gene model. Without a gene model, 3% to 9% of non-junctions reads became multiple mapped reads. Thus, it is rare for a non-junction read to become unmapped or mapped alternatively. However, the mapping of junction reads was strongly impacted by the gene models (see Figure 3C). Without using a gene model, an average of 53% of junction reads remained mapped to the same genomic regions, 30% of failed to map to any genomic region, and 10–15% of them mapped alternatively. Such alternative mappings are generally inferior compared to their corresponding mapping results using a gene model [20]. Similar to non-junction reads, an average of 5% of junction reads were mapped to more than one location without using a gene model. As shown in Figure 3C, more uniquely-mapped junction reads became multiple mapped reads in RefGene and/or UCSC than in Ensembl when the sequence reads were aligned to the reference genome without the use of gene models.

### The impact of gene model choice on gene quantification

**Figure 4:**
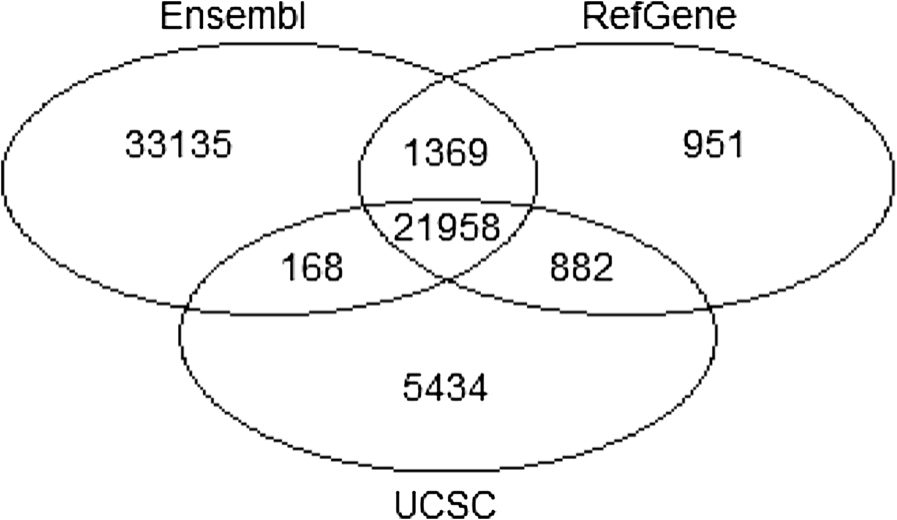
**The overlap and intersection among RefGene,** **UCSC,** **and Ensembl annotations.** In general, different annotations have very high overlaps: there are 21,598 common genes shared by all three gene models. RefGene has the fewest unique genes, while more than 50% of genes in Ensembl are unique.

Different gene identifiers are used in different annotation databases; therefore, we mapped those database-specific identifiers into the unique HGNC gene symbols from the HUGO Gene Nomenclature Committee when comparing their gene quantification results across the different gene models originating from these databases. Considering that annotations are more or less incomplete in these databases, we only focused on common genes. The Venn diagram in Figure 4 showed the overlap and intersection of RefGene, UCSC, and Ensembl annotations. Clearly RefGene has fewest unique genes, while more that 50% of genes in Ensembl are unique. In general, the different annotations have very high overlaps: 21,598 common genes are shared by all three gene annotations.

**Figure 5:**
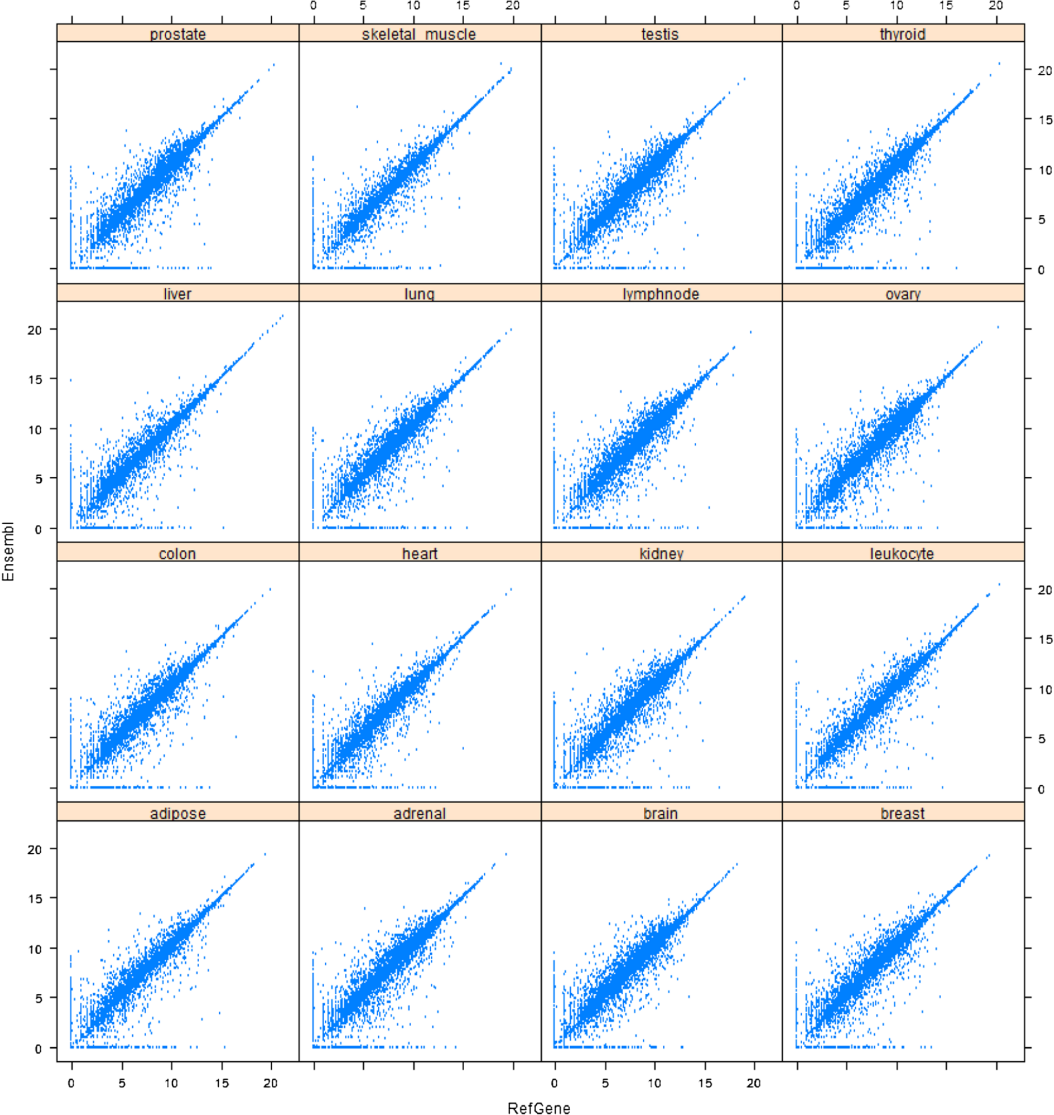
**The correlation of gene quantification results between RefGene and Ensembl.** Both x and y-axes represent Log2(count + 1). Although the majority of genes have highly consistent or nearly identical expression levels, there are many genes whose quantification results are dramatically affected by the choice of a gene model.

To investigate the impact of different gene models on gene quantification results, we focused on this set of 21,598 common genes. The overall correlation between RefGene and Ensembl was shown in Figure 5. Both x and y-axes represented log2(count + 1). For all genes, 1 was added to the counts to avoid a logarithmic error for those genes with zero counts. Ideally, we should get identical numbers of mapped reads for all common genes, regardless of the choice of a gene model; however, this was clearly not the case. Although the majority of genes had highly consistent or nearly identical expression levels, there were a significant number of genes whose quantification results were dramatically affected by the choice of a gene model. As shown in Figure 5, there were many genes for which the number of reads mapped to them was 0 in one gene model, but many in others.

To quantify the concordance between RefGene and Ensembl annotations, we first calculated the ratio of mapped read for each gene. For a given gene, we defined the raw read counts in RefGene and Ensembl annotations as #C1 and #C2, respectively. To prevent division by 0, 1 was added to all raw read counts before the ratios were calculated. The adjusted counts were denoted as #C1’ (=#C1 + 1) and #C2’ (=#C2 + 1), respectively. The ratio was calculated as Max(#C1’,#C2’)/Min(#C1’,#C2’). Therefore the calculated ratio was always equal or greater than 1. The distribution of ratios was summarized in Table 1 (read length = 75 bp). Among the 21,958 common genes, about 20% of genes had no expression at all in both annotations. Identical counts were obtained for only 16.3% of genes. Approximately 28.1% of genes’ expression levels differed by 5% or higher, and among them, 9.3% of genes (equivalent to 2038) differed by 50% or greater. As shown in Table 1 and Figure 5, the choice of a gene model had a large impact on gene quantification. The concordance between UCSC and RefGene annotation was reported in Additional file 1: Table S7 (read length = 75 bp). Compared with Ensembl, UCSC had a much better concordance with RefGene, in terms of the gene quantification results. 38.3% of genes had identical read counts, much higher than the 16.3% between Ensembl and RefGene. The percentage of genes with expression levels differing by 5% or more was only 11.3%, which was much less than the corresponding 28% between Ensembl and RefGene. Furthermore, only 3.24% of genes‘ quantification results differed by 50% or greater, which was lower than the 9.3% between Ensembl and RefGene.

**Table 1:** **The distribution of the ratio of read counts between RefGene and Ensembl annotations (read length** = **75 bp)**

**Sample**	**No Expr**	**Same**	**1.05**	**1.10**	**1.20**	**1.50**	**2**	**5**	**10**	**100**
Adipose	19.97	16.53	26.16	19.64	14.51	8.81	5.65	1.96	0.94	0.16
Adrenal	16.92	14.04	36.18	27.09	19.07	11.28	7.14	2.45	1.24	0.24
Brain	16.79	15.22	32.94	24.91	17.95	10.78	6.73	2.29	1.08	0.20
Breast	18.04	15.22	29.63	22.21	16.06	9.80	6.52	2.38	1.19	0.20
Colon	20.50	17.41	25.85	19.43	14.30	8.95	6.10	2.30	1.17	0.19
Heart	21.23	16.43	26.39	20.10	14.39	8.88	5.47	1.73	0.82	0.19
Kidney	18.86	16.08	28.88	21.50	15.51	9.55	6.40	2.55	1.30	0.26
Leukocyte	29.53	17.37	20.03	15.29	11.62	7.58	5.37	2.47	1.33	0.26
Liver	24.60	19.16	23.20	17.43	12.84	8.24	5.42	2.00	1.02	0.15
Lung	19.65	16.46	29.22	21.35	15.07	9.09	6.15	2.61	1.43	0.24
Lymph node	20.94	16.79	31.74	24.16	17.21	10.26	6.65	2.69	1.44	0.24
Ovary	16.90	13.42	31.46	23.30	16.72	10.23	6.63	2.31	1.13	0.20
Prostate	18.21	16.29	28.33	21.14	15.17	9.43	6.51	2.49	1.27	0.23
Skeletal muscle	29.60	23.48	18.65	14.40	10.73	6.88	4.81	2.34	1.39	0.21
Testis	10.15	13.35	31.35	22.57	15.84	9.35	5.92	2.08	1.05	0.28
Thyroid	17.41	14.25	30.08	22.23	15.88	9.39	5.88	1.97	1.03	0.24
**Average**	19.96	16.34	28.13	21.05	15.18	9.28	6.09	2.29	1.18	0.22

**Note**: Column “**No Expr**” represents the percentage of genes that do not express at all in both annotations. Column “**Same**” denotes the percentage of genes that have the same number of reads mapped to them in both gene models. The number in each cell after the column “**Same**” corresponds to the percentage of genes whose ratio is equal or greater than the threshold represented by the number.

**Figure 6:**
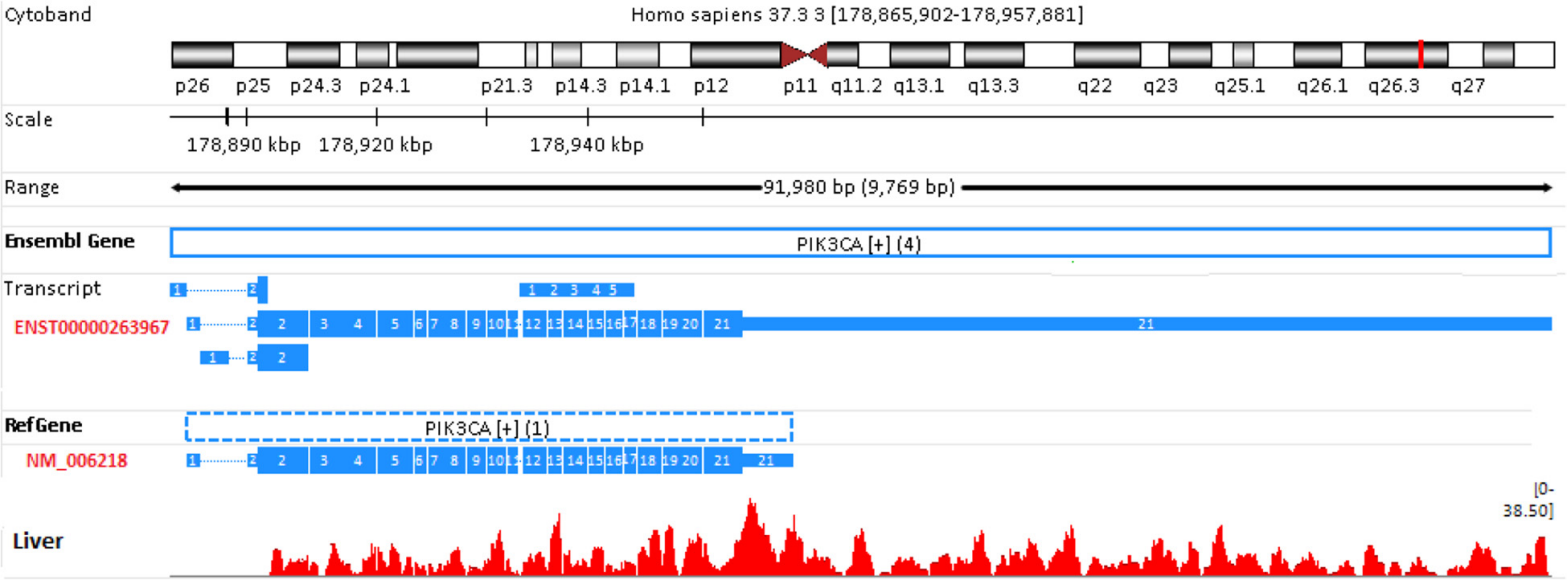
**The different gene definitions for PIK3CA give rise to differences in gene quantification.** PIK3CA in the Ensembl annotation is much longer than its definition in RefGene, explaining why there are 1094 reads mapped to PIK3CA in Ensembl, while only 492 reads are mapped in RefGene. The PIK3CA gene definition in Ensembl seems more accurate than the one in RefGene, based upon the mapping profile of sequence reads.

Why does the choice of a gene model have so dramatic an effect on gene quantification? Below, we chose a few extreme or representative cases to provide possible explanations. In the liver sample, the expression levels for these exemplary genes for both Ensembl and RefGene were summarized in Table 2 (read length = 75 bp). PIK3CA (phosphatidylinositol-4,5-bisphosphate 3-kinase, catalytic subunit alpha) uses ATP to phosphorylate PtdIns, PtdIns4P, and PtdIns(4,5)P2. In the liver sample, there were 1094 reads mapped to PIK3CA in Ensembl annotation, while only 492 reads were mapped in RefGene. The PIK3CA gene definition in both Ensembl and RefGene, and the mapping profile of RNA-Seq reads were shown in Figure 6. Clearly, the difference in gene definition gives rise to the observed discrepancy in quantification. In Ensembl, there are three isoforms for PIK3CA, and the longest isoform is ENST00000263967. The total length of this transcript is 9653 bp, comprising 21 exons, with a very long exon #21 (6000 bp, chr3: 178,951,882-178,957,881). In RefGene, PIK3CA has only one transcript named NM_006218. This transcript is 3909 bp long with a very short exon #21 (only 616 bp, located at chr 3:178,951,882-178,952,497). The definition of PIK3CA gene in Ensembl seems more accurate than the one in RefGene, based upon the mapping profile of the sequence reads. Likewise, the difference in read counts for gene EGFR and SLC30A1 in Ensembl and RefGene mainly results from the gene definition difference (Additional file 1: Figures S4 and S5).

**Table 2:** **Gene definitions and quantification results for certain exemplary genes in the liver tissue sample (read length = 75 bp)**

**Model**	**Gene**	**Counts**	**Transcript**	**Strand**	**Chromosome**	**Start**	**End**	**Length**
**Ensembl**	PIK3CA	**1094**	4	+	3	178,865,902	178,957,881	9411
	EGFR	**6644**	11	+	7	55,086,714	55,324,313	12961
	SLC30A1	**9755**	1	-	1	211,744,910	211,752,084	5474
	PIGY	**0**	1	-	4	89,442,724	89,442,940	217
	PYURF	**1799**	1	-	4	89,442,136	89,444,964	1361
	LUZP6	**0**	1	-	7	135,612,022	135,612,198	177
	MTPN	**2618**	2	-	7	135,611,509	135,662,101	3775
	PECAM1	**0**	9	-	HG183_PATCH	62,399,863	62,491,136	4671
**RefGene**	PIK3CA	**492**	1	+	3	178,866,311	178,952,497	3709
	EGFR	**2248**	4	+	7	55,086,725	55,275,031	6571
	SLC30A1	**1636**	1	-	1	211,748,381	211,752,099	2018
	PIGY	**1175**	1	-	4	89,442,129	89,444,952	1356
	PYURF	**1175**	1	-	4	89,442,129	89,444,952	1356
	LUZP6	**1908**	1	-	7	135,611,503	135,662,204	3884
	MTPN	**1908**	1	-	7	135,611,503	135,662,204	3884
	PECAM1	**1068**	1	-	17	62,396,777	62,407,083	4453

**Figure 7:**
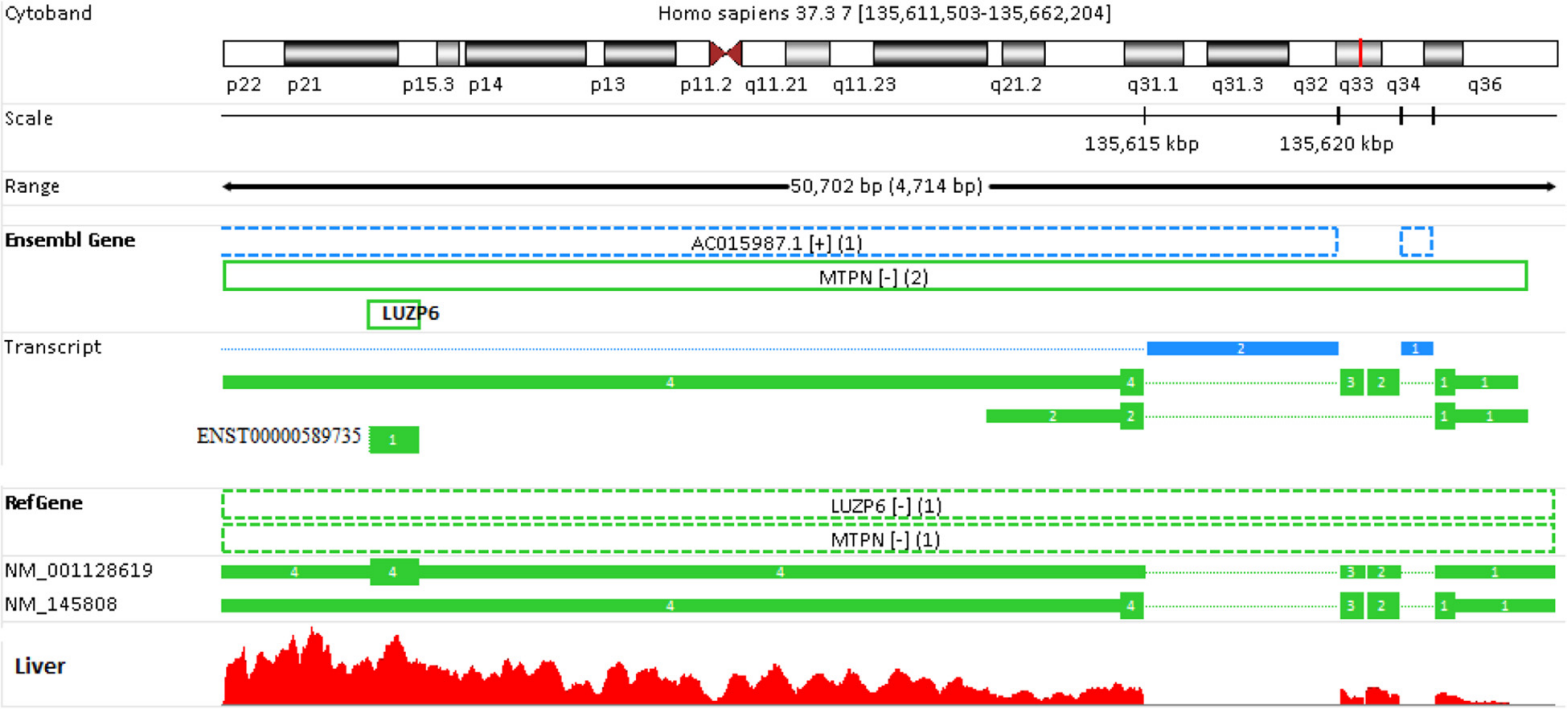
**The different gene definitions for LUZP6.** In the Ensembl annotation, LUZP6 is only 177 bp long, and it is completely within another gene, MTPN. As a result, all sequence reads originating from LUZP6 are assigned to MTPN instead. In RefGene, LUZP6 and MTPN are derived from the same genomic region, and both encode exactly the same mRNA, though the protein coding sequences are different. Therefore, all reads mapped to this region are equally distributed between these two genes.

Figure 7 shows another example of a remarkably different gene model defined in Ensembl versus that in RefGene. In RefGene, a bi-cistronic transcript encodes the products of both the MTPN (myotrophin) and LUZP6 (leucine zipper protein 6) genes, which are located on chromosome 7. All mapped reads are equally distributed to these two genes. The mature transcript is 3884 bp in RefGene. However, in Ensembl, LUZP6 is only 177 bp long, and is completely within MTPN. As a consequence, all reads mapped to the overlapping region are assigned to MTPN only because LUZP6 does not have any unique reads mapped to it, which explains why the read count for LUZP6 was 0 when Ensembl annotation was chosen. Likewise, the difference in gene definition (see Additional file 1: Figure S6) can explain the quantification results for PIGY/PYURF in Table 2. The gene PIGY in Ensembl is only 217 bp long and overlaps completely with PYURF (PIGY Upstream Reading Frame). Thus, all reads mapped to the region of PIGY are assigned to gene PYURF, while no read is given to PIGY. In RefGene, PIGY and PYURF encode exactly the same mRNA, although the translated protein sequences are different. Thus, all reads mapped to PIGY/PYURF are equally distributed to these two genes. The gene PECAM1 is another interesting example. It is located on chromosome 17 in the RefGene model. In Ensembl, however, this gene is located on chromosome HG183_PATCH: 62,399,863-62,491,136. HG183_PATCH is not included in the human genome GRCH37.3 at all, explaining why zero reads mapped to gene PECAM1 using Ensembl annotation.

### The effect of gene models on differential analysis

**Figure 8:**
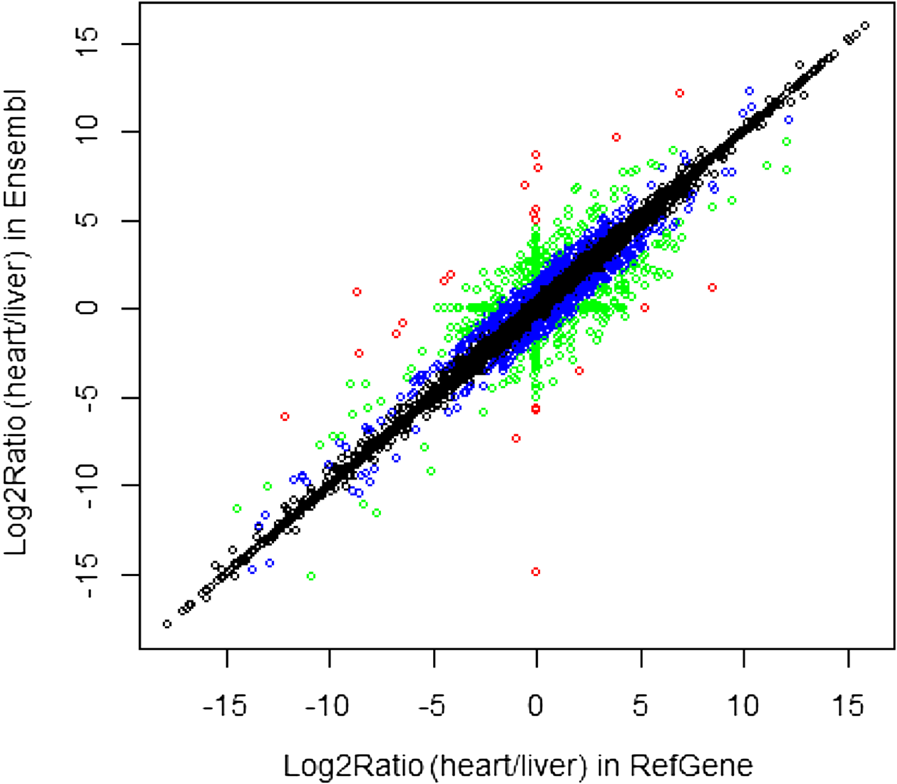
**The correlation of the calculated Log2Ratio** **(heart/** **liver)** **between RefGene and Ensembl.** The green, blue, and red points indicate corresponding absolute difference between the two Log2Ratios that were greater than 1, 2, or 5, respectively. Although the majority of genes have highly consistent expression changes, there are many genes that are remarkably affected by the choice of different gene models.

Generally, RNA-Seq differential analysis requires biological replicates. However, we analyzed single samples from 16 different tissues. To demonstrate the effect of gene models on differential analysis, the fold changes between heart and liver samples were calculated using RefGene and Ensembl annotations. The correlation of the calculated Log2Ratio (liver/heart) was depicted in Figure 8. The graph should show a perfect diagonal line if the choice of a gene model has no effect on differential analysis. Although the majority of genes have highly consistent or comparable expression changes, there are a number of genes whose ratios are dramatically affected by the choice of a gene model. Interestingly, some genes have a very high fold change in one gene model, but no change at all in another gene model. Evidently, the choice of a gene model has an effect on the downstream differential expression analysis, in addition to gene quantification.

### The effect of a gene model on mapping is read length dependent

All the analysis results for the dataset with a 50-bp read length were reported in the supplementary tables and figures. Intuitively, the shorter a read, the more likely it is to map to multiple locations. As a result, the percentage of uniquely mapped reads decreases, and the percentage of multiple-mapping reads increases. No matter which gene model was used for mapping, this observation held true; for example if we compare Additional file 1: Table S1 with Additional file 1: Table S2, and/or Additional file 1: Table S3 with Additional file 1: Table S4. Thus, the mapping fidelity for a sequence read increases with its length, and this is especially true for junction reads. As demonstrated in Figure 3C and Additional file 1: Table S5, when the read length was 75 bp, an average of 53% of junction reads remained mapped to the same genomic regions when mapped without gene annotation. However, this percentage dropped to 42% when the read length was 50 bp long (Additional file 1: Figure S3C and Additional file 1: Table S6). Thus, the effect of a gene model on the mapping of junction reads is significantly influenced by read length.

In the meantime, the relative abundance of junction reads is heavily determined by read length.

According to Figure 3A and Additional file 1: Table S5, on average, roughly 23% of sequence reads were junction reads when the read length was 75 bp. The percentage of junction reads dropped to 16% when the read length was 50 bp (see Additional file 1: Figure S3A and Additional file 1: Table S6). This is explained by the fact that the longer the read, the more likely that it spans more than one exon. As sequencing technology evolves, the read length will become longer and longer. Consequently, more junction reads will be generated by short-gun sequencing technologies. Therefore, the need to incorporate genome annotation in the read mapping process will greatly increase.

### Which genome annotation to choose for gene quantification?

In practice, there is no simple answer to this question, and it depends on the purpose of the analysis. In this paper, we demonstrated that the choice of a gene model has an effect on the quantification results. Previously, we compared the gene quantification results when RefGene and Ensembl annotations were used. Among 25,958 common genes, the expressions of 2038 genes (i.e., 9.3%) differed by 50% or more when choosing one annotation over the other. Such a large difference frequently results from the gene definition differences in the annotations. Genes with the same HUGO symbol in different gene models can be defined as completely different genomic regions. When choosing an annotation database, researchers should keep in mind that no database is perfect and some gene annotations might be inaccurate or entirely wrong.

Wu *et al*. [27] suggested that when conducting research that emphasizes reproducible and robust gene expression estimates, a less complex genome annotation, such as RefGene, might be preferred. When conducting more exploratory research, a more complex genome annotation, such as Ensembl, should be chosen. Based upon our experience of RNA-Seq data analysis, we recommend using RefGene annotation if RNA-Seq is used as a replacement for a microarray in transcriptome profiling. For human samples, Affymetrix GeneChip HT HG-U133+ PM arrays are one of the most popular microarray platforms for transcriptome profiling, and the genes covered by this chip overlap with RefGene very well, according to Zhao *et al*. [6] h. Despite the fact that Ensembl R74 contains 63,677 annotated gene entries, only 22,810 entries (roughly one third) correspond to protein coding genes. There are 17,057 entries representing various types of RNAs, including rRNA (566), snoRNA (1549), snRNA (2067), miRNA (3361), misc_RNA (2174), and lincRNA (7340). There are 15,583 pseudogenes in Ensembl R74. For most RNA-Seq sequencing projects, only mRNAs are presumably enriched and sequenced, and there is no point in mapping sequence reads to RNAs such as miRNAs or lincRNAs. Ensembl R74 contains 819 processed transcripts that were generated by reverse transcription of an mRNA transcript with subsequent reintegration of the cDNA into the genome, and are usually not actively expressed. In this scenario, a read truly originating from an active mRNA can be mapped to the processed transcript or mapped to the processed transcript only, which is especially true for junction reads. Consequently, the true expression for the corresponding mRNA may be underestimated. Another downside of using a larger annotation database is calculation of adjusted p values, because the adjustment of the raw p value to allow for multiple testing is mainly determined by the number of genes in the model. If genes of interest are defined inconsistently across different annotations, it is recommended that the RNA-Seq dataset is analyzed using different gene models.

## Conclusions

RNA-Seq has become increasingly popular in transcriptome profiling. Acquiring transcriptome expression profiles requires researchers to choose a genome annotation for RNA-Seq data analysis. In this paper, we assessed the impact of gene models on the mapping of junction and non-junction reads, and compared the impact of genome annotation choice on gene quantification and differential analysis. To fairly assess the impact of a gene model on RNA-Seq read mapping, we devised a two-stage mapping protocol, in which sequence reads that could not be mapped to a reference transcriptome were filtered out, and the remaining reads were mapped to the reference genome with and without the use of a gene model in the mapping step. Our protocol ensured that only those reads compatible with a gene model were used to evaluate the role of a genome annotation in RNA-Seq data analysis.

Ensembl annotates more genes than RefGene and UCSC. On average, 95% of non-junction reads were mapped to exactly the same genomic location without the use of a gene model. However, only an average of 53% junction reads remained mapped to the same genomic regions. About 30% of junction reads failed to be mapped without the assistance of a gene model, while 10–15% mapped alternatively. It is also demonstrated that the effect of a gene model on the mapping of sequence reads is significantly influenced by read length. The mapping fidelity for a sequence read increases with its length. When the read length was reduced from 75 bp to 50 bp, the percentage of junction reads that remained mapped to the same genomic regions dropped from 53% to 42% without the assistance of gene annotation.

There are 21,958 common genes among RefGene, Ensembl, and UCSC annotations. Using the dataset with the read length of 75 bp, we compared the gene quantification results in RefGene and Ensembl annotations, and obtained identical counts for an average of 16.3% (about one sixth) of genes. Twenty percent of genes are not expressed, and thus have zero counts in both annotations. About 28.1% of genes showed expression levels that differed by 5% or higher; of these, the relative expression levels for 9.3% of genes (equivalent to 2038) differed by 50% or greater. The case studies revealed that the difference in gene definitions caused the observed inconsistency in gene quantification.

## Methods

The Human Body Map 2.0 Project, using Illumina sequencing, generated RNA-Seq data for 16 different human tissues (adipose, adrenal, brain, breast, colon, heart, kidney, leukocyte, liver, lung, lymph node, ovary, prostate, skeletal muscle, testis, and thyroid) and is accessible from ArrayExpress (accession number E-MTAB-513). To demonstrate the impact of read length on analysis results, we created a new dataset in which each original 75-bp long sequence read was trimmed to 50 bp. The same analysis protocol described below was applied to both datasets. The RefGene, Ensembl, and UCSC annotation files in GTF format were downloaded from the UCSC genome browser.

Primary sequencing reads were first mapped to reference transcriptome and the human reference genome GRCH37.3 using Omicsoft sequence aligner (OSA) [16]. Benchmarked with existing methods such as Tophat and others, OSA improves mapping speed 4–10 fold, with better sensitivity and fewer false positives. When a gene model is used in conjunction with a reference genome, by default, OSA maps RNA-Seq reads in three consecutive steps: (1) all reads are mapped to the reference transcriptome; (2) for mapped reads with mismatches, OSA aligns them with the reference genome and chooses the best hits; and (3) for unmapped reads, OSA maps them to reference genome. OSA can be finely controlled, and step #1 could be run alone if only those reads that could be mapped to a reference transcriptome were desired.

**Figure 9:**
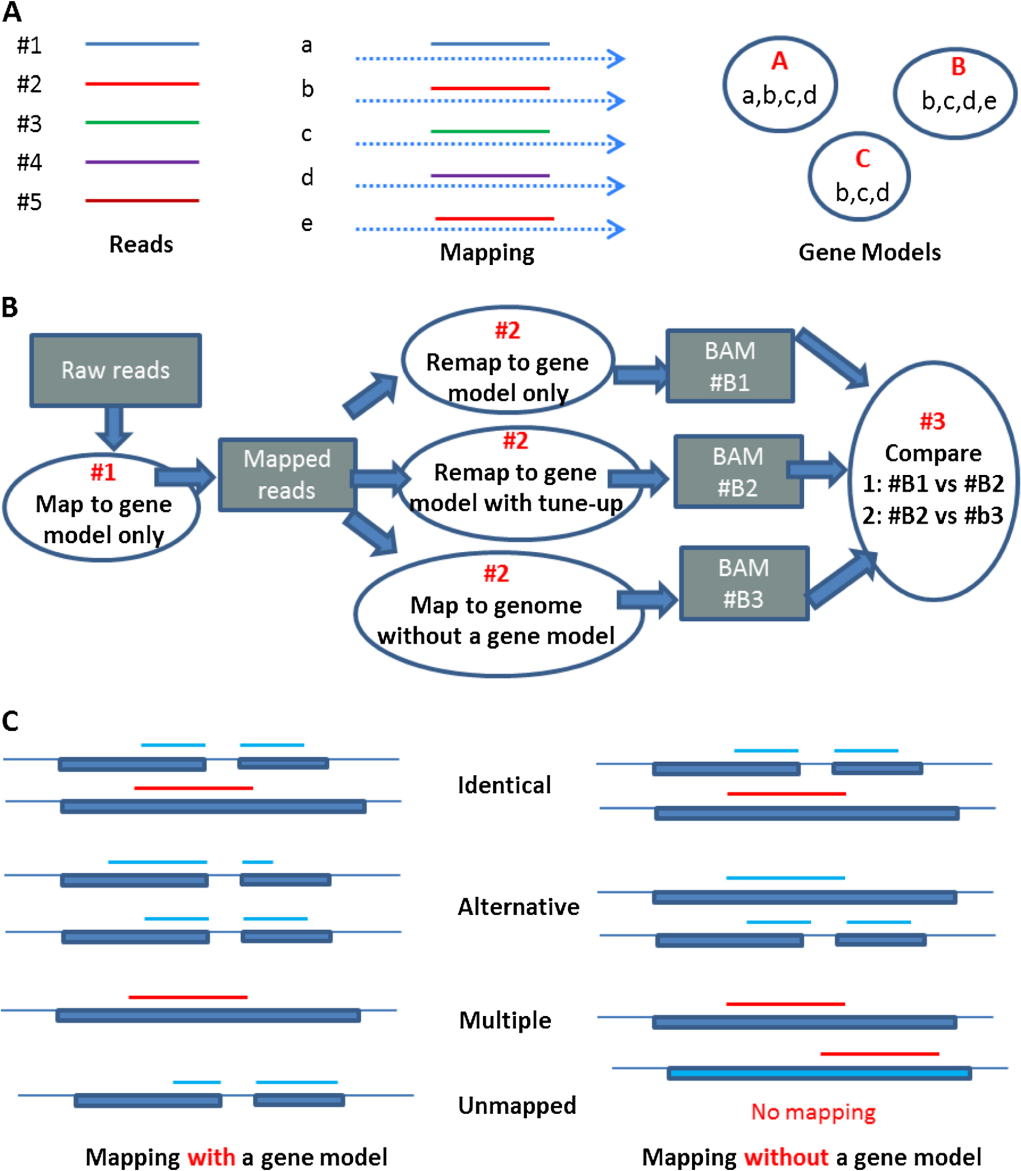
**Analysis protocol. (A)** The mapping result for a sequence read that is gene model dependent, where none of the gene models are complete; **(B)** “two-stage” mapping protocol: at Stage #1, all RNA-Seq reads are mapped to a reference transcriptome only, and then only the mapped reads are saved into a new FASTQ file; at Stage #2, those remaining reads are mapped to the genome with and without the use of a gene model in the mapping step; **(C)** The protocol for classifying uniquely mapped sequence reads into four categories, i.e., “Identical”, “Alternative”, “Multiple” and “Unmapped” (or Fail).

As shown in Figure 9A, the mapping result of a sequence read is gene model dependent. For instance, read #2 could be uniquely mapped to gene #b if the gene model #A was chosen in the mapping step. However, this read became a multipl-mapped read when either gene model #B or #C was chosen, because it could be mapped to genes #b and #e equally well. None of the gene models are complete; therefore, we devised a two-stage mapping protocol to investigate the effect of a gene model on RNA-Seq data analysis (Figure 9B). At Stage #1, all RNA-Seq reads were mapped to a reference transcriptome only, and then only mapped reads are saved into a new FASTQ file. At Stage #2, the remaining reads were mapped to a genome using three different mapping modes: (1) “transcriptome only”, every read was mapped to either a unique or multiple locations in annotated regions; (2) “transcriptome only + tune up”, similar to “transcriptome only”, but for those mapped reads with mismatches, they were mapped to genome as well, and the best hits were selected from the mapping results. For ties, the read was mapped to reference transcriptome; and (3) “None”, reads were mapped to reference genome directly, without the use of a gene model in the mapping step. According to our results (unpublished), there was only a small difference (less than 0.5% of reads) between “transcriptome only” and “transcriptome only + tune up” modes. Therefore, to quantify the effect of a gene model on mapping of RNA-Seq reads, we only compared the results from “transcriptome only” mode with those from the “None” mode in Stage #2.

Accordingly, the effect of a gene model on RNA-Seq read mapping could be characterized and quantified by comparing the mapping results in different mapping modes. We focused on those uniquely mapped reads in the “transcriptome only” mode, and divided them into four categories (Figure 9C) according to their mapping results without a gene annotation in mapping step: (1) “Identical”, the same alignment results were obtained regardless of the use of a gene model; (2) “Alternative”, the read still mapped but mapped differently. It turns out that the majority of reads in this category were junction reads. A junction read could be either mapped as a non-junction read, or remain mapped as a junction read but with different start, end, and splicing positions; (3) “Multiple”, a uniquely mapped read became a multiple-mapped one. When a read is mapped across the whole reference genome, it is more likely to be mapped to multiple locations; and (4) “Unmapped”, i.e., a read could not be mapped to anywhere in the genome without the assistance of a gene model. Nearly all reads in this category were junction reads.

## Additional file

Additional file 1: Tables S1 and S2. Report the mapping summaries for all 16 tissue samples in different mapping modes when the read Length is 75 bp and 50 bp, respectively. **Tables S3** and **S4** contain the re-mapping summaries corresponding to the read length of 75 bp and 50 bp, respectively. Reads not compatible with a gene model in “transcriptome only” mode are filtered out first prior to re-mapping. **Tables S5** and **S6** summarize the impact of the usage of a gene model on the mapping of junction and non-junction reads in all 16 tissue samples. The corresponding read lengths are 75 bp and 50 bp, respectively. **Table S7** reports the distribution of the ratio of read counts between RefGene and UCSC annotations. The read length is 75 bp. **Figure S1** is the plot of the read mapping summary for all 16 tissue samples in “transcriptome only” and “transcriptome + genome” mapping modes. The read Length is 50 bp. **Figure S2** shows the impact of a gene model on the mapping of reads when the read Length is 50 bp. **Figure S3** quantifies the impact of a gene model on the mapping of junction and non-junction reads. The read Length is 50 bp. **Figure S4** shows the EGFR quantification difference between Ensembl and RefGene results from the difference in gene definition. **Figure S5** highlights the gene definition difference for SLC30A1 in Ensembl and RefGene. The exons region defined in Ensembl is almost 3 times as long as in RefGene. **Figure S6** shows the gene definition difference for PIGY in Ensembl and RefGene, and accordingly explains why the gene quantification results dramatically differ from each other.
