# Supplementary material for: A comprehensive evaluation of ensembl, RefSeq, and UCSC annotations in the context of RNA-seq read mapping and gene quantification
Source: BMC Genomics. 2015 Feb 18;16(1):97. doi: 10.1186/s12864-015-1308-8 (PMC4339237; doi:10.1186/s12864-015-1308-8)
Supplement: Additional file 1: Tables S1 and S2. — Report the mapping summaries for all 16 tissue samples in different mapping modes when the read Length is 75 bp and 50 bp, respectively. Tables S3 and S4 contain the re-mapping summaries corresponding to the read length of 75 bp and 50 bp, respectively. Reads not compatible with a gene model in “transcriptome only” mode are filtered out first prior to re-mapping. Tables S5 and S6 summarize the impact of the usage of a gene model on the mapping of junction and non-junction reads in all 16 tissue samples. The corresponding read lengths are 75 bp and 50 bp, respectively. Table S7 reports the distribution of the ratio of read counts between RefGene and UCSC annotations. The read length is 75 bp. Figure S1 is the plot of the read mapping summary for all 16 tissue samples in “transcriptome only” and “transcriptome + genome” mapping modes. The read Length is 50 bp. Figure S2 shows the impact of a gene model on the mapping of reads when the read Length is 50 bp. Figure S3 quantifies the impact of a gene model on the mapping of junction and non-junction reads. The read Length is 50 bp. Figure S4 shows the EGFR quantification difference between Ensembl and RefGene results from the difference in gene definition. Figure S5 highlights the gene definition difference for SLC30A1 in Ensembl and RefGene. The exons region defined in Ensembl is almost 3 times as long as in RefGene. Figure S6 shows the gene definition difference for PIGY in Ensembl and RefGene, and accordingly explains why the gene quantification results dramatically differ from each other. [file 12864_2015_1308_MOESM1_ESM.pdf]

Supplementary Table 1 Mapping summaries for all 16 tissue samples in different mapping modes (Read Length = 75 bp, “Uniq” means “Unique”)

Mapping Mode	Tissue	Total read#	Ensembl (%)			UCSC (%)			RefGene (%)			None (%)		
			Uniq	Non-uniq	Unmap	Uniq	Non-uniq	Unmap	Uniq	Non-uniq	Unmap	Uniq	Non-uniq	Unmap
A: transcriptome+genome	adipose	67973941	87.54	7.08	5.39	85.73	8.86	5.41	85.87	8.71	5.42	77.70	12.26	10.04
	adrenal	68177365	86.54	6.96	6.50	86.72	6.71	6.58	86.70	6.60	6.70	78.70	10.56	10.74
	brain	48351853	88.19	5.60	6.21	88.27	5.49	6.24	88.35	5.38	6.27	82.19	7.37	10.44
	breast	67344979	88.04	6.42	5.53	86.60	7.84	5.56	86.72	7.72	5.57	79.29	10.82	9.89
	colon	70480743	85.93	8.29	5.78	88.05	6.15	5.80	88.22	5.96	5.82	79.50	10.49	10.01
	heart	62477941	87.05	8.53	4.42	86.46	9.09	4.45	86.54	8.99	4.47	80.47	10.63	8.90
	kidney	70586055	85.79	7.66	6.54	86.02	7.41	6.57	86.13	7.28	6.59	79.54	10.03	10.43
	leukocyte	73747241	88.44	7.76	3.80	93.12	3.05	3.83	93.37	2.75	3.88	81.27	8.77	9.96
	liver	60602560	87.06	7.85	5.09	87.70	7.19	5.11	88.19	6.76	5.05	79.30	9.10	11.60
	lung	74157861	88.26	7.42	4.32	91.08	4.55	4.36	91.17	4.35	4.49	81.51	9.01	9.48
	lymphnode	74296479	83.20	8.73	8.07	86.18	5.58	8.25	85.04	5.79	9.17	76.73	9.91	13.36
	ovary	71922658	86.40	7.88	5.72	89.53	4.72	5.75	89.76	4.46	5.78	79.39	9.77	10.84
	prostate	76591270	88.67	7.57	3.76	92.53	3.68	3.79	92.80	3.38	3.81	82.37	8.79	8.84
	skeletal_muscle	74935505	90.05	6.67	3.28	91.37	5.32	3.31	91.48	5.19	3.33	83.86	7.41	8.74
	testis	74891611	88.68	6.22	5.10	89.86	4.99	5.16	89.98	4.82	5.20	81.08	8.01	10.91
	thyroid	72231690	88.04	5.88	6.09	90.10	3.77	6.13	90.25	3.59	6.16	81.21	6.99	11.80
B: transcriptome only	adipose	67973941	80.81	6.71	12.48	65.11	1.30	33.58	64.83	1.11	34.06			
	adrenal	68177365	69.38	6.29	24.34	61.65	1.70	36.65	59.57	1.04	39.39			
	brain	48351853	75.20	5.03	19.77	62.57	1.12	36.30	62.00	0.94	37.06			
	breast	67344979	78.41	5.94	15.65	64.60	1.39	34.01	64.12	1.16	34.72			
	colon	70480743	80.69	7.95	11.36	63.58	2.21	34.21	63.43	2.04	34.53			
	heart	62477941	79.83	7.94	12.23	51.91	0.94	47.14	51.70	0.84	47.46			
	kidney	70586055	77.07	7.03	15.90	58.96	1.21	39.83	58.52	1.04	40.44			
	leukocyte	73747241	83.96	7.58	8.46	83.34	1.55	15.11	83.05	1.18	15.77			
	liver	60602560	82.84	7.62	9.54	71.47	3.74	24.80	71.74	3.28	24.98			
	lung	74157861	80.98	7.15	11.87	77.72	2.38	19.90	76.37	1.77	21.87			
	lymphnode	74296479	73.79	8.29	17.92	70.14	3.72	26.14	61.98	1.28	36.74			
	ovary	71922658	75.74	7.40	16.87	72.53	1.56	25.91	72.05	1.17	26.78			
	prostate	76591270	81.78	7.30	10.93	78.95	1.56	19.49	78.56	1.18	20.26			
	skeletal_muscle	74935505	86.43	6.38	7.18	68.59	1.29	30.12	67.82	1.14	31.03			
	testis	74891611	80.06	5.77	14.16	71.36	1.64	26.99	70.99	1.41	27.60			
	thyroid	72231690	78.46	5.47	16.07	73.36	1.51	25.13	72.73	1.26	26.01			

Supplementary Table 2 Mapping summaries for all 16 tissue samples (Read Length = 50 bp)

Mode	Tissue	Total read#	Ensembl			UCSC			RefGene			None		
			Uniq	Non-uniq	Unmap	Uniq	Non-uniq	Unmap	Uniq	Non-uniq	Unmap	Uniq	Non-uniq	Unmap
transcriptome+genome	adipose	67973941	85.68	10.40	3.92	84.90	11.17	3.93	85.09	10.96	3.96	76.13	16.34	7.53
	adrenal	68177365	85.73	10.08	4.19	86.90	8.88	4.22	87.06	8.62	4.32	78.14	14.43	7.43
	brain	48351853	87.47	8.31	4.22	88.22	7.53	4.26	88.36	7.35	4.29	81.61	10.72	7.67
	breast	67344979	86.57	9.42	4.00	85.93	10.04	4.02	86.12	9.84	4.04	77.89	14.68	7.43
	colon	70480743	83.18	12.62	4.20	86.97	8.82	4.22	87.15	8.62	4.24	76.84	15.76	7.40
	heart	62477941	83.58	13.39	3.04	83.39	13.54	3.06	83.67	13.24	3.09	77.23	16.11	6.66
	kidney	70586055	83.90	11.47	4.63	84.85	10.49	4.65	85.10	10.23	4.67	77.67	14.70	7.63
	leukocyte	73747241	86.63	10.71	2.65	92.75	4.58	2.68	93.12	4.16	2.72	79.90	12.70	7.40
	liver	60602560	85.18	11.23	3.59	86.34	10.05	3.61	86.79	9.62	3.59	78.42	12.75	8.83
	lung	74157861	86.54	10.37	3.09	90.48	6.40	3.12	90.75	6.04	3.21	80.20	12.80	7.00
	lymphnode	74296479	80.90	12.98	6.13	85.06	8.78	6.16	84.59	8.58	6.83	75.17	14.97	9.86
	ovary	71922658	85.20	10.68	4.12	89.59	6.26	4.15	89.94	5.88	4.18	78.32	13.56	8.12
	prostate	76591270	86.89	10.44	2.67	92.03	5.28	2.70	92.41	4.86	2.72	80.84	12.61	6.55
	skeletal_muscle	74935505	87.09	10.49	2.41	89.34	8.21	2.44	89.51	8.02	2.46	81.46	11.90	6.64
	testis	74891611	87.29	8.99	3.72	89.47	6.75	3.77	89.66	6.53	3.81	80.29	11.53	8.18
	thyroid	72231690	87.19	8.39	4.41	90.28	5.26	4.45	90.49	5.03	4.49	80.77	10.29	8.95
transcriptome only	adipose	67973941	79.28	9.99	10.73	65.95	2.22	31.83	65.73	1.98	32.29			
	adrenal	68177365	69.06	9.41	21.53	62.84	3.10	34.06	60.96	2.08	36.96			
	brain	48351853	74.76	7.64	17.60	64.01	1.85	34.14	63.49	1.64	34.86			
	breast	67344979	77.38	8.94	13.68	65.48	2.36	32.16	65.07	2.03	32.90			
	colon	70480743	78.26	12.15	9.58	65.25	3.62	31.13	65.06	3.46	31.48			
	heart	62477941	76.79	12.79	10.41	52.77	1.78	45.44	52.74	1.66	45.60			
	kidney	70586055	75.61	10.85	13.55	60.23	2.25	37.51	59.95	2.04	38.01			
	leukocyte	73747241	82.38	10.53	7.09	83.75	2.64	13.61	83.60	2.13	14.27			
	liver	60602560	81.23	10.95	7.82	71.71	4.89	23.40	71.94	4.50	23.56			
	lung	74157861	79.54	10.11	10.36	78.01	3.69	18.30	76.91	2.77	20.32			
	lymphnode	74296479	71.86	12.56	15.58	70.02	6.46	23.52	63.01	2.16	34.83			
	ovary	71922658	74.88	10.18	14.94	73.33	2.51	24.16	72.99	1.97	25.03			
	prostate	76591270	80.27	10.14	9.58	79.40	2.55	18.06	79.16	2.02	18.82			
	skeletal_muscle	74935505	83.79	10.20	6.01	69.20	2.21	28.58	68.56	2.04	29.40			
	testis	74891611	79.06	8.47	12.46	72.31	2.55	25.13	72.01	2.25	25.73			
	thyroid	72231690	77.99	7.97	14.04	74.42	2.36	23.22	73.87	2.04	24.09			

Supplementary Table 3 Re-mapping summaries for all 16 samples (Read Length = 75 bp). Reads not compatible with a gene model in “transcriptome only” mode are filtered out first prior to mapping

Model	Tissue	Reads	Trancriptome only		Transcriptome + tune up		None		
			Uniq	Non-uniq	Uniq	Non-uniq	Uniq	Non-uniq	Unmap
Ensembl	adipose	59490660	92.33	7.67	92.33	7.67	81.10	13.59	5.31
	adrenal	51584273	91.69	8.31	91.68	8.32	81.35	13.07	5.58
	brain	38793525	93.73	6.27	93.71	6.29	86.24	8.50	5.27
	breast	56803305	92.96	7.04	92.95	7.05	82.58	12.26	5.16
	colon	62470965	91.03	8.97	91.03	8.97	83.78	11.45	4.77
	heart	54839276	90.95	9.05	90.95	9.05	83.46	11.44	5.10
	kidney	59363034	91.64	8.36	91.63	8.37	84.21	11.18	4.61
	leukocyte	67505294	91.72	8.28	91.73	8.27	83.90	9.37	6.72
	liver	54818447	91.58	8.42	91.60	8.40	83.03	9.79	7.19
	lung	65357107	91.88	8.12	91.89	8.11	84.25	9.91	5.84
	lymphnode	60979931	89.90	10.10	89.94	10.06	82.09	11.54	6.37
	ovary	59791468	91.10	8.90	91.08	8.92	82.66	11.19	6.15
	prostate	68223533	91.81	8.19	91.80	8.20	84.74	9.57	5.70
	skeletal_muscle	69552798	93.12	6.88	93.12	6.88	86.46	7.67	5.87
	testis	64284318	93.27	6.73	93.26	6.74	84.43	8.83	6.74
	thyroid	60622324	93.49	6.51	93.47	6.53	85.35	7.86	6.79
RefGene	adipose	44823454	98.31	1.69	98.53	1.47	86.17	6.85	6.98
	adrenal	41320239	98.28	1.72	98.20	1.79	85.06	8.34	6.60
	brain	30431357	98.50	1.50	98.68	1.31	88.92	4.48	6.61
	breast	43961453	98.22	1.78	98.35	1.65	86.99	6.41	6.60
	colon	46143763	96.89	3.11	97.95	2.05	84.65	8.97	6.38
	heart	32824395	98.41	1.59	98.67	1.33	87.14	4.45	8.41
	kidney	42037686	98.26	1.74	98.53	1.47	87.50	6.07	6.43
	leukocyte	62116747	98.60	1.40	98.66	1.34	84.32	8.48	7.20
	liver	45461616	95.63	4.37	95.67	4.33	83.84	7.45	8.71
	lung	57942475	97.74	2.26	97.74	2.26	85.43	8.25	6.32
	lymphnode	46997211	97.98	2.02	97.72	2.28	84.76	9.09	6.16
	ovary	52662361	98.41	1.59	98.38	1.61	84.24	8.88	6.88
	prostate	61073715	98.53	1.47	98.55	1.45	85.49	8.24	6.28
	skeletal_muscle	51680168	98.34	1.66	98.67	1.33	87.64	4.55	7.81
	testis	54221537	98.05	1.95	98.21	1.79	85.98	6.20	7.83
	thyroid	53446031	98.30	1.70	98.33	1.67	86.15	6.27	7.58
UCSC	adipose	45146557	98.04	1.96	98.24	1.76	86.16	6.88	6.96
	adrenal	43191486	97.31	2.69	97.31	2.69	84.69	8.77	6.54
	brain	30799133	98.23	1.77	98.41	1.59	88.88	4.54	6.58
	breast	44443645	97.89	2.11	98.01	1.99	86.95	6.50	6.55
	colon	46371858	96.63	3.37	97.59	2.41	84.61	9.01	6.39
	heart	33024094	98.21	1.79	98.44	1.56	87.14	4.46	8.40
	kidney	42469360	97.99	2.01	98.25	1.75	87.50	6.10	6.40
	leukocyte	62606247	98.18	1.82	98.23	1.77	84.29	8.50	7.21
	liver	45574213	95.03	4.97	95.09	4.91	83.93	7.46	8.61
	lung	59400151	97.03	2.97	97.08	2.92	85.16	8.48	6.36
	lymphnode	54876890	94.96	5.04	95.04	4.96	82.29	10.85	6.86
	ovary	53287663	97.89	2.11	97.90	2.10	84.23	8.92	6.85
	prostate	61661130	98.06	1.94	98.11	1.89	85.52	8.23	6.25
	skeletal_muscle	52363510	98.15	1.85	98.44	1.56	87.70	4.55	7.75
	testis	54674934	97.75	2.25	97.90	2.10	85.93	6.25	7.82
	thyroid	54078618	97.98	2.02	98.02	1.98	86.17	6.28	7.55

Supplementary Table 4 Re-mapping summaries for all 16 samples (Read Length = 50 bp). Reads not compatible with a gene model in "transcriptome only" mode are filtered out first prior to mapping

Model	Tissue	Reads	Trancriptome only		Transcriptome + tune up		None		
			Uniq	Non-uniq	Uniq	Non-uniq	Uniq	Non-uniq	Unmap
Ensembl	adipose	60676666	88.81	11.19	88.85	11.15	78.14	17.81	4.05
	adrenal	53497968	88.01	11.99	88.14	11.86	78.48	17.38	4.14
	brain	39843773	90.73	9.27	90.74	9.25	83.64	12.17	4.19
	breast	58129274	89.64	10.36	89.70	10.30	79.64	16.39	3.97
	colon	63724914	86.56	13.44	86.58	13.42	79.57	16.89	3.54
	heart	55972392	85.72	14.28	85.73	14.26	78.66	17.30	4.04
	kidney	61024330	87.45	12.55	87.50	12.50	80.29	16.24	3.47
	leukocyte	68518286	88.67	11.33	88.70	11.29	81.46	13.43	5.12
	liver	55865660	88.12	11.88	88.14	11.85	80.81	13.51	5.69
	lung	66475349	88.73	11.27	88.79	11.21	81.72	13.92	4.37
	lymphnode	62721277	85.12	14.88	85.29	14.71	78.51	17.06	4.43
	ovary	61173476	88.04	11.96	88.07	11.92	79.98	15.31	4.71
	prostate	69249229	88.78	11.22	88.82	11.18	82.13	13.57	4.30
	skeletal_muscle	70430620	89.15	10.85	89.17	10.83	83.17	12.33	4.50
	testis	65558025	90.32	9.68	90.35	9.65	82.35	12.55	5.10
	thyroid	62086619	90.73	9.27	90.77	9.23	83.29	11.43	5.28
RefGene	adipose	46024218	97.08	2.92	97.40	2.60	84.16	10.55	5.29
	adrenal	42978657	96.70	3.30	96.64	3.35	82.50	12.55	4.95
	brain	31494140	97.48	2.52	97.82	2.17	87.47	7.34	5.20
	breast	45191287	96.97	3.03	97.20	2.80	84.94	10.00	5.06
	colon	48293615	94.95	5.05	96.52	3.47	81.48	13.90	4.62
	heart	33989300	96.94	3.06	97.56	2.43	85.73	7.70	6.57
	kidney	43757206	96.70	3.30	97.31	2.68	85.32	9.90	4.78
	leukocyte	63225280	97.52	2.48	97.65	2.35	82.24	12.30	5.46
	liver	46325187	94.11	5.89	94.20	5.80	83.25	9.89	6.86
	lung	59086063	96.52	3.48	96.59	3.41	83.35	11.89	4.75
	lymphnode	48415416	96.68	3.32	96.31	3.68	81.86	13.49	4.64
	ovary	53917032	97.37	2.63	97.38	2.61	81.89	12.86	5.26
	prostate	62173876	97.51	2.49	97.63	2.37	83.37	11.91	4.72
	skeletal_muscle	52904839	97.11	2.89	97.71	2.29	86.30	7.78	5.92
	testis	55618330	96.97	3.03	97.24	2.75	84.63	9.48	5.89
	thyroid	54828276	97.31	2.69	97.40	2.59	84.60	9.52	5.88
UCSC	adipose	46338250	96.75	3.25	97.08	2.92	84.22	10.50	5.28
	adrenal	44956873	95.30	4.70	95.43	4.57	82.15	12.98	4.88
	brain	31843405	97.19	2.81	97.53	2.46	87.51	7.30	5.19
	breast	45683508	96.53	3.47	96.79	3.21	84.94	10.04	5.02
	colon	48536492	94.75	5.25	96.23	3.77	81.52	13.85	4.63
	heart	34085303	96.73	3.27	97.36	2.64	86.07	7.33	6.60
	kidney	44107409	96.39	3.61	97.01	2.98	85.51	9.71	4.77
	leukocyte	63712467	96.94	3.06	97.10	2.90	82.24	12.29	5.47
	liver	46421300	93.62	6.38	93.72	6.27	83.38	9.80	6.81
	lung	60585919	95.48	4.52	95.65	4.35	83.06	12.18	4.75
	lymphnode	56820194	91.56	8.44	91.77	8.23	78.84	16.33	4.83
	ovary	54549161	96.69	3.31	96.76	3.24	81.90	12.86	5.24
	prostate	62760619	96.89	3.11	97.06	2.94	83.41	11.88	4.70
	skeletal_muscle	53514788	96.90	3.10	97.50	2.50	86.46	7.66	5.88
	testis	56067586	96.59	3.41	96.88	3.11	84.62	9.49	5.89
	thyroid	55459454	96.92	3.08	97.04	2.95	84.65	9.49	5.86

Supplementary Table 5 The impact of the usage of a gene model on the mapping of junction and non-junction reads in all 16 tissue samples (Note: Read Length = 75 bp)

Model	Tissue	Total Reads	Category (%)		Non-junction Reads (%)				Junction Reads (%)			
			NonJunc	Junc	Identical	Alternative	Multiple	Fail	Identical	Alternative	Multiple	Fail
Ensembl	adipose	54927843	81.02	18.98	91.02	0.00	8.95	0.02	57.43	11.07	1.94	29.56
	adrenal	47292893	80.27	19.73	92.17	0.01	7.79	0.03	56.39	11.49	2.34	29.78
	brain	36352401	83.76	16.24	96.37	0.00	3.59	0.03	56.73	8.67	0.94	33.66
	breast	52798273	82.23	17.77	92.17	0.00	7.80	0.03	57.33	10.67	1.80	30.20
	colon	56866240	82.60	17.40	95.60	0.00	4.38	0.02	56.97	11.37	2.35	29.30
	heart	49873780	83.37	16.63	96.36	0.00	3.62	0.02	56.50	8.98	1.54	32.99
	kidney	54393727	83.99	16.01	95.54	0.01	4.43	0.02	56.98	10.87	1.56	30.60
	leukocyte	61919320	76.25	23.75	96.87	0.01	3.08	0.04	56.70	10.95	2.20	30.15
	liver	50212520	78.11	21.89	97.26	0.00	2.71	0.03	55.46	8.06	2.68	33.80
	lung	60058055	79.24	20.76	96.30	0.01	3.67	0.03	56.38	11.32	3.06	29.24
	lymphnode	54844293	79.05	20.95	96.38	0.01	3.59	0.03	54.04	11.42	2.73	31.81
	ovary	54460236	77.80	22.20	95.14	0.01	4.82	0.04	57.16	11.17	2.16	29.51
	prostate	62632183	78.58	21.42	96.62	0.01	3.35	0.02	57.80	11.58	2.31	28.31
	skeletal_mus	64769324	78.86	21.14	98.02	0.00	1.96	0.02	57.88	11.20	1.68	29.24
	testis	59951007	77.64	22.36	95.90	0.00	4.07	0.02	56.68	10.47	1.44	31.41
	thyroid	56665319	76.46	23.54	96.89	0.01	3.07	0.03	57.81	10.57	1.51	30.12
UCSC	adipose	44350820	74.40	25.60	94.57	0.01	5.39	0.03	52.59	14.35	5.95	27.10
	adrenal	42029104	75.50	24.50	93.55	0.01	6.40	0.04	50.73	15.25	7.35	26.67
	brain	30308240	79.49	20.51	96.71	0.00	3.24	0.04	53.62	11.02	3.45	31.90
	breast	43560918	76.79	23.21	95.08	0.01	4.89	0.03	53.02	13.71	5.32	27.95
	colon	45256454	75.63	24.37	93.04	0.01	6.93	0.02	50.99	15.12	7.64	26.25
	heart	32509770	73.49	26.51	96.73	0.00	3.24	0.03	54.21	10.84	3.30	31.64
	kidney	41726991	77.65	22.35	95.25	0.01	4.71	0.04	53.02	13.64	4.83	28.52
	leukocyte	61500411	73.27	26.73	93.08	0.01	6.87	0.05	50.59	14.44	8.07	26.90
	liver	43334428	73.85	26.15	96.63	0.00	3.33	0.04	53.69	9.84	3.91	32.56
	lung	57667842	76.19	23.81	94.26	0.01	5.70	0.03	51.01	14.83	7.74	26.42
	lymphnode	52153249	75.75	24.25	93.58	0.01	6.38	0.03	48.71	15.05	7.73	28.51
	ovary	52168619	73.93	26.07	92.92	0.01	7.04	0.04	50.64	15.00	8.18	26.18
	prostate	60498557	75.19	24.81	93.53	0.01	6.43	0.03	51.45	15.50	7.85	25.20
	skeletal_mus	51544182	72.00	28.00	96.76	0.00	3.22	0.02	54.76	13.80	3.77	27.67
	testis	53527201	73.45	26.55	95.25	0.00	4.72	0.03	53.00	13.25	4.38	29.37
	thyroid	53008906	73.12	26.88	95.21	0.01	4.74	0.03	53.87	13.31	4.74	28.08
RefGene	adipose	44165802	74.22	25.78	94.47	0.01	5.50	0.03	52.22	14.51	6.31	26.96
	adrenal	40578418	74.84	25.16	93.36	0.01	6.59	0.04	50.47	15.48	7.99	26.06
	brain	30030853	79.30	20.70	96.63	0.00	3.33	0.04	53.29	11.26	3.75	31.71
	breast	43235214	76.53	23.47	95.00	0.01	4.96	0.03	52.49	13.96	5.69	27.87
	colon	45197955	75.47	24.53	92.95	0.01	7.02	0.02	50.51	15.24	8.23	26.01
	heart	32387323	73.40	26.60	96.61	0.00	3.35	0.03	53.94	11.04	3.48	31.54
	kidney	41420902	77.43	22.57	95.14	0.01	4.81	0.04	52.66	13.80	5.20	28.34
	leukocyte	61282842	73.07	26.93	92.92	0.01	7.02	0.05	50.11	14.58	8.68	26.63
	liver	43495259	73.35	26.65	96.57	0.00	3.39	0.04	52.18	10.32	4.85	32.66
	lung	56632569	75.88	24.12	94.10	0.01	5.86	0.03	50.70	15.04	8.44	25.83
	lymphnode	45925280	75.19	24.81	93.20	0.01	6.76	0.04	50.58	15.98	8.58	24.85
	ovary	51811217	73.60	26.40	92.73	0.01	7.22	0.04	50.10	15.13	8.88	25.88
	prostate	60186691	74.90	25.10	93.35	0.01	6.62	0.03	50.89	15.59	8.58	24.93
	skeletal_mus	50993874	71.68	28.32	96.65	0.00	3.33	0.02	54.49	13.98	3.96	27.57
	testis	53251574	73.31	26.69	95.13	0.00	4.84	0.03	52.63	13.44	4.74	29.19
	thyroid	52554153	72.87	27.13	95.08	0.01	4.87	0.03	53.49	13.51	5.09	27.92

Supplementary Table 6 The impact of the usage of a gene model on the mapping of junction and non-junction reads in all 16 tissue samples (Note: Read Length = 50 bp)

Model	Tissue	Total Reads	Category (%)		Non-junction Reads (%)				Junction Reads (%)			
			NonJunc	Junc	Identical	Alternative	Multiple	Fail	Identical	Alternative	Multiple	Fail
Ensembl	adipose	53908913	87.37	12.63	90.49	0.02	9.45	0.04	45.43	16.88	3.39	34.31
	adrenal	47153945	87.04	12.96	91.56	0.02	8.35	0.07	44.85	17.79	4.15	33.21
	brain	36153828	89.16	10.84	95.47	0.01	4.44	0.08	44.78	13.62	1.78	39.82
	breast	52141615	88.18	11.82	91.35	0.02	8.58	0.05	45.72	16.30	3.03	34.95
	colon	55174995	88.35	11.65	94.55	0.01	5.40	0.04	45.34	17.11	4.10	33.44
	heart	47987647	88.55	11.45	95.35	0.01	4.60	0.05	44.49	14.03	2.16	39.32
	kidney	53397449	89.28	10.72	94.38	0.02	5.56	0.05	45.73	16.73	2.79	34.75
	leukocyte	60777766	84.28	15.72	95.82	0.02	4.08	0.08	44.99	16.40	3.89	34.73
	liver	49242653	85.47	14.53	96.73	0.01	3.20	0.06	43.96	12.42	2.99	40.62
	lung	59025503	86.26	13.74	95.36	0.02	4.57	0.05	45.03	17.27	4.41	33.29
	lymphnode	53495624	86.00	14.00	95.68	0.02	4.23	0.06	43.59	16.96	5.85	33.60
	ovary	53877042	85.42	14.58	94.15	0.02	5.76	0.08	45.04	16.69	3.91	34.35
	prostate	61506724	85.88	14.12	95.65	0.02	4.28	0.05	45.79	17.50	4.07	32.65
	skeletal_mus	62802332	85.70	14.30	97.15	0.01	2.80	0.04	46.51	16.59	2.91	33.99
	testis	59229707	85.13	14.87	95.13	0.01	4.80	0.06	45.46	15.91	2.63	36.00
	thyroid	56356419	84.37	15.63	95.99	0.02	3.93	0.06	45.79	16.02	2.78	35.42
UCSC	adipose	44985823	82.93	17.07	91.86	0.02	8.06	0.06	40.18	20.42	9.00	30.40
	adrenal	42901746	83.61	16.39	90.84	0.02	9.05	0.09	38.74	21.57	10.99	28.70
	brain	31058092	86.34	13.66	94.52	0.01	5.36	0.10	41.14	16.86	5.33	36.67
	breast	44217845	84.50	15.50	92.47	0.02	7.44	0.07	40.96	19.65	8.01	31.37
	colon	46705583	84.00	16.00	89.19	0.01	10.75	0.05	38.89	21.02	11.40	28.70
	heart	33184504	82.36	17.64	94.60	0.01	5.32	0.07	41.63	16.74	4.78	36.85
	kidney	42790119	85.20	14.80	92.56	0.02	7.35	0.07	41.23	20.06	7.37	31.34
	leukocyte	61865260	81.98	18.02	90.15	0.02	9.74	0.09	38.44	20.09	11.78	29.69
	liver	43508122	82.71	17.29	95.38	0.01	4.54	0.08	41.71	14.86	5.09	38.33
	lung	57950037	84.01	15.99	91.63	0.02	8.29	0.05	39.21	20.96	10.78	29.05
	lymphnode	52141440	83.52	16.48	91.08	0.02	8.82	0.07	37.28	20.87	12.61	29.25
	ovary	52780920	82.51	17.49	89.86	0.02	10.04	0.09	38.17	20.53	12.16	29.14
	prostate	60916059	83.33	16.67	90.74	0.02	9.19	0.05	39.01	21.56	11.63	27.81
	skeletal_mus	52176620	81.28	18.72	94.37	0.01	5.59	0.04	42.62	20.00	6.20	31.19
	testis	54319565	82.28	17.72	92.85	0.01	7.06	0.07	41.24	19.22	6.91	32.63
	thyroid	53819394	81.96	18.04	92.79	0.02	7.12	0.07	41.34	19.30	7.40	31.97
RefGene	adipose	44826838	82.80	17.20	91.61	0.02	8.30	0.07	39.82	20.62	9.40	30.17
	adrenal	41534239	83.21	16.79	90.23	0.02	9.64	0.11	38.39	21.87	11.59	28.15
	brain	30807057	86.20	13.80	94.30	0.01	5.57	0.11	40.80	17.18	5.64	36.37
	breast	43924092	84.32	15.68	92.22	0.02	7.68	0.08	40.48	19.87	8.43	31.22
	colon	46614303	83.86	16.14	89.02	0.01	10.92	0.05	38.43	21.14	12.06	28.38
	heart	33159444	82.33	17.67	94.05	0.01	5.86	0.08	41.35	16.98	5.02	36.66
	kidney	42579997	85.06	14.94	92.17	0.02	7.73	0.08	40.82	20.25	7.84	31.08
	leukocyte	61738417	81.82	18.18	89.80	0.02	10.07	0.10	37.94	20.22	12.57	29.27
	liver	43636330	82.36	17.64	95.13	0.01	4.77	0.08	40.55	15.23	5.98	38.24
	lung	57071192	83.79	16.21	91.20	0.02	8.72	0.06	38.77	21.15	11.58	28.50
	lymphnode	46628836	83.39	16.61	89.76	0.02	10.14	0.09	37.98	22.16	12.58	27.28
	ovary	52505302	82.29	17.71	89.42	0.02	10.46	0.10	37.68	20.68	12.87	28.76
	prostate	60697493	83.12	16.88	90.35	0.02	9.57	0.06	38.45	21.61	12.51	27.42
	skeletal_mus	51692759	81.05	18.95	94.13	0.01	5.81	0.05	42.32	20.20	6.49	30.99
	testis	54083911	82.18	17.82	92.61	0.01	7.30	0.08	40.86	19.44	7.30	32.40
	thyroid	53403512	81.79	18.21	92.51	0.02	7.39	0.08	40.98	19.55	7.74	31.73

Supplementary Table 7 The distribution of the ratio of read counts between RefGene and UCSC annotations (Read Length = 75 bp)

Sample	No Expr	Same	1.05	1.10	1.20	1.50	2	5	10	100
adipose	19.97	38.27	10.59	7.71	5.48	3.22	1.99	0.65	0.35	0.09
adrenal	16.92	36.54	14.79	10.32	6.92	3.92	2.37	0.73	0.41	0.08
brain	16.79	38.32	13.20	9.30	6.40	3.69	2.13	0.70	0.38	0.04
breast	18.04	37.54	12.07	8.60	5.75	3.32	2.12	0.81	0.40	0.09
colon	20.50	39.72	10.37	7.51	5.22	3.10	1.97	0.80	0.47	0.07
heart	21.23	38.48	10.38	7.48	5.22	3.03	1.80	0.56	0.31	0.09
kidney	18.86	38.66	11.64	8.05	5.49	3.17	2.01	0.78	0.48	0.10
leukocyte	29.53	36.90	8.02	5.90	4.27	2.77	1.85	0.90	0.47	0.09
liver	24.60	40.26	9.22	6.55	4.65	2.92	1.87	0.64	0.31	0.06
lung	19.65	39.12	11.86	8.12	5.49	3.08	2.00	0.87	0.50	0.11
lymphnode	20.94	38.31	13.13	9.25	6.24	3.52	2.15	0.88	0.51	0.13
ovary	16.90	35.98	12.25	8.49	5.98	3.38	2.15	0.78	0.43	0.07
prostate	18.21	38.07	11.40	8.13	5.59	3.35	2.13	0.89	0.47	0.12
skeletal_muscle	29.60	42.49	7.54	5.60	4.07	2.58	1.71	0.87	0.52	0.08
testis	10.15	38.38	12.57	8.59	5.82	3.36	1.94	0.68	0.37	0.07
thyroid	17.41	36.14	12.28	8.55	5.77	3.38	1.99	0.76	0.36	0.09
**Average**	19.96	38.32	11.33	8.01	5.52	3.24	2.01	0.77	0.42	0.09

Note: Column "**No Expr**" represents the percentage of genes that do not express at all in both annotations. Column "**Same**" denotes the percentage of genes that have the same number of reads mapped to them in both gene models. The number in each cell after column "Same" corresponds to the percentage of genes whose ratio is above the corresponding threshold in that column.

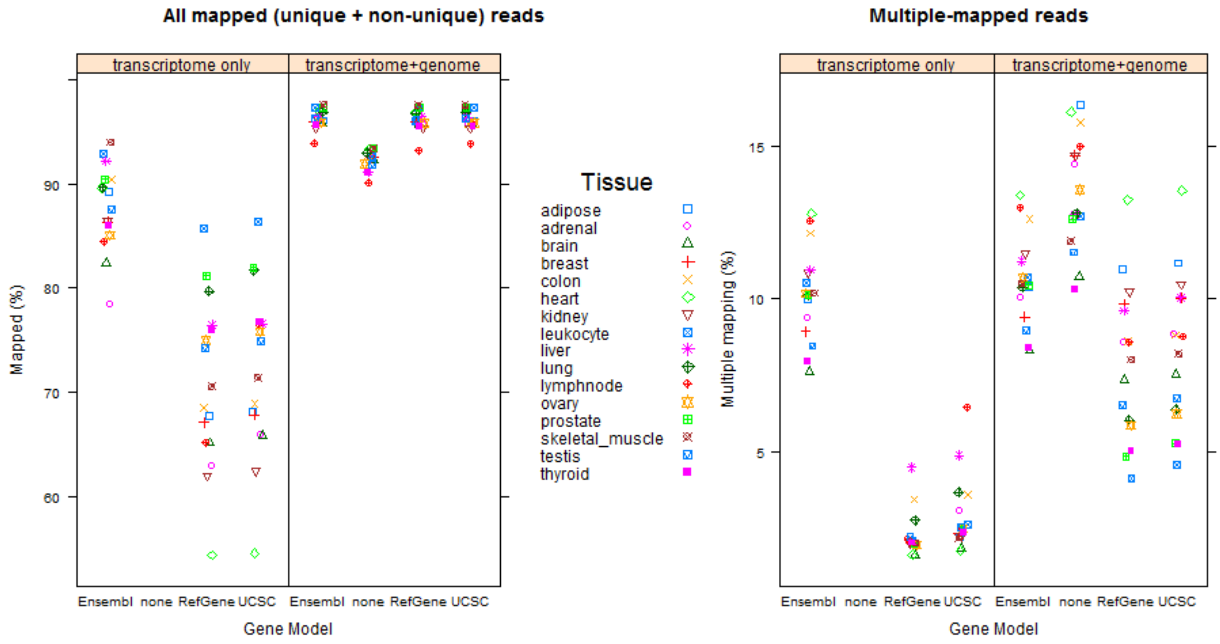

Supplementary Figure 1. The read mapping summary for 16 tissue samples in “transcriptome only” and “transcriptome+genome” mapping modes (note: Read Length = 50 bp). In “transcriptome only” mode, more reads are mapped in Ensembl than RefGene and UCSC (left panel), and also more read become multiple mapped ones in Ensembl than RefGene and UCSC (right panel).

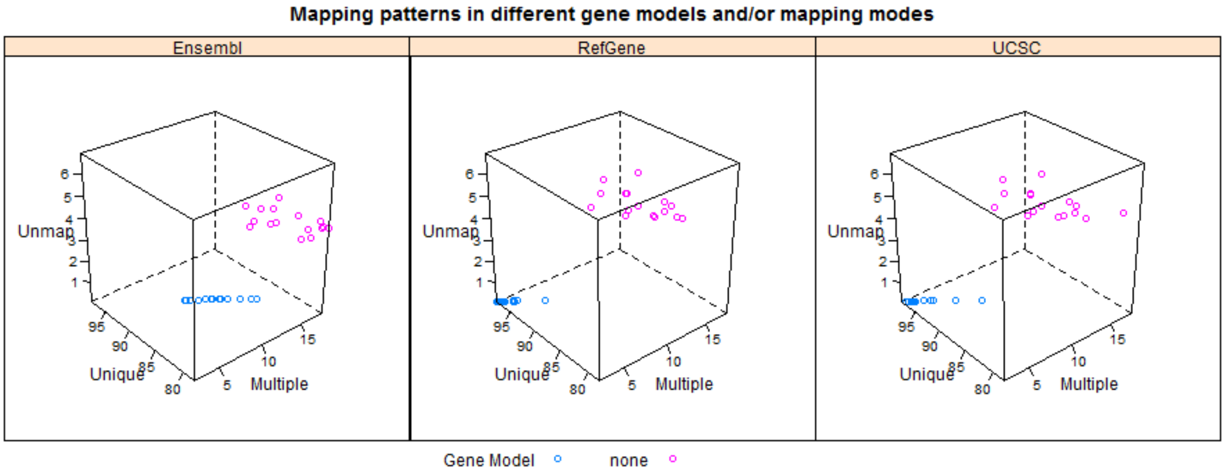

Supplementary Figure 2. The impact of a gene model on the mapping summaries for all 16 tissue samples (Note: Read Length = 50 bp). The RefGene and UCSC consistently have the highest percentage of uniquely mapped reads; while the percentage of non-uniquely mapped reads is much higher in Ensembl. Without a gene model (samples colored in pink), some reads become unmapped, and the average percentage of unmapped reads about 5%.

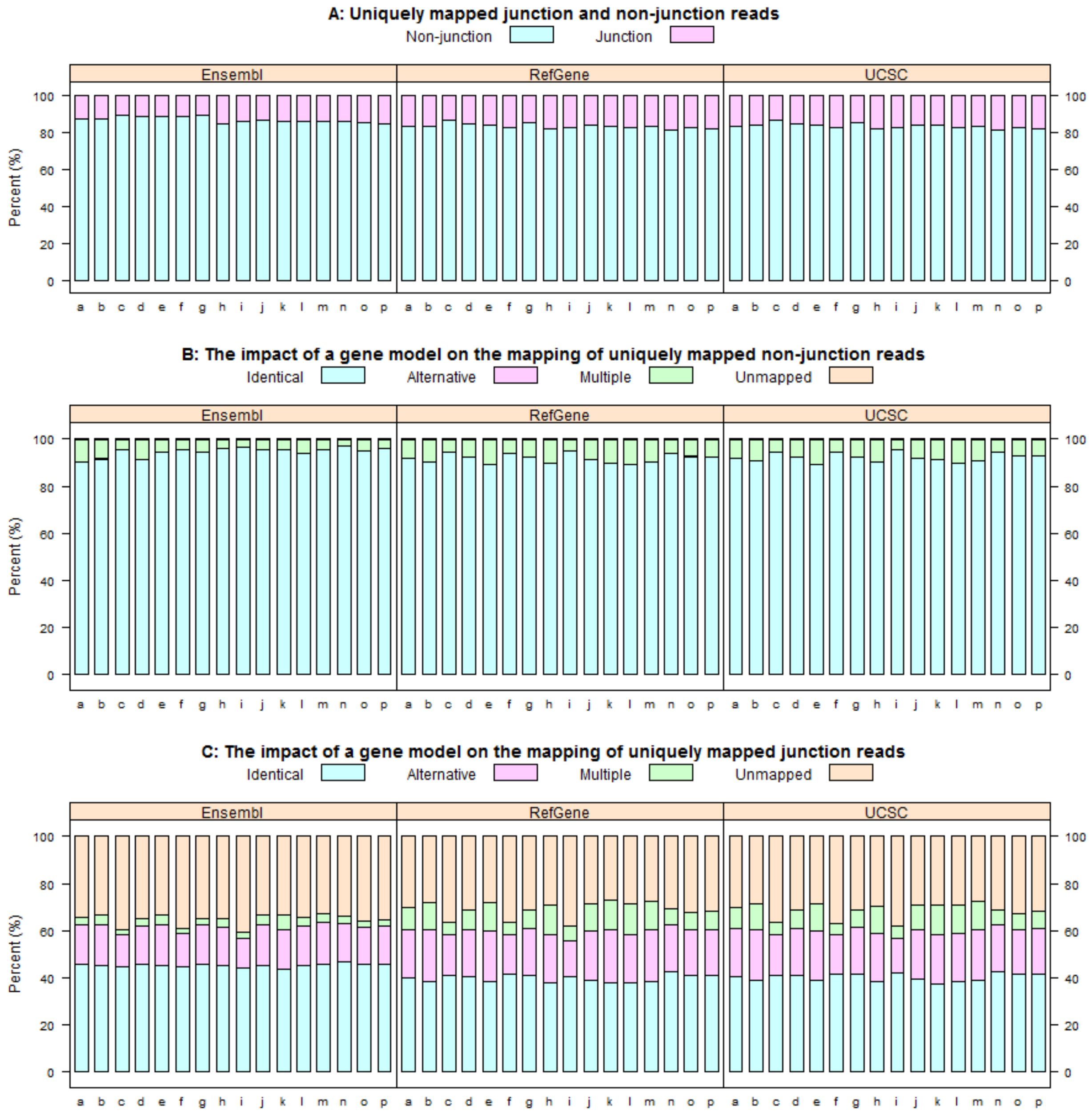

Supplementary Figure 3. The impact of a gene model on RNA-Seq read mapping (Note: Read Length = 50 bp). (A) composition of mapped reads: roughly 14% are junction ones, and the rest 86% are non-junction ones; (B) impact on non-junctions reads: on average, 93% remains mapped to exactly the same genomic location, whilst ~7% of reads becomes multiple mapping ones; (C) impact on junctions reads: only an average of ~42% junction reads remain mapped to the same genomic regions without the assistance of a gene model in mapping. About 33% of junction reads fail to be mapped, while 18% mapped alternatively. Note: all 16 tissue sample names are denoted as follows: **a**: adipose; **b**: adrenal, **c**: brain; **d**: breast; **e**: colon; **f**: heart; **g**: kidney; **h**: leukocyte; **i**: liver; **j**: lung; **k**: lymphnode; **l**: ovary; **m**: prostate; **n**: skeletal_muscle; **o**: testis; and **p**: thyroid.

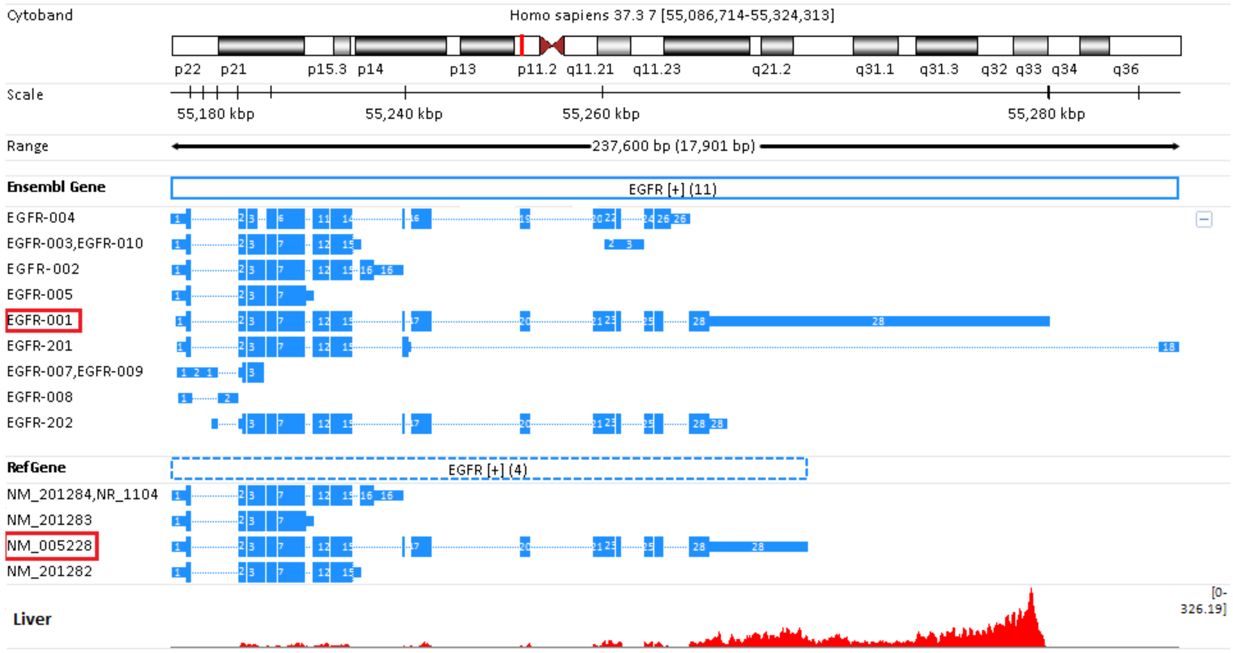

Supplementary Figure 4. The gene definitions for EGFR. In Ensembl annotation, there are 11 transcripts while in RefGene, the number of transcripts is 4. The main expressed isoform (i.e. EGFR-001) in Ensembl is much longer than its counterpart NM_005228 in RefGene. As a result, more reads are counted for EGFR in Ensembl than in RefGene.

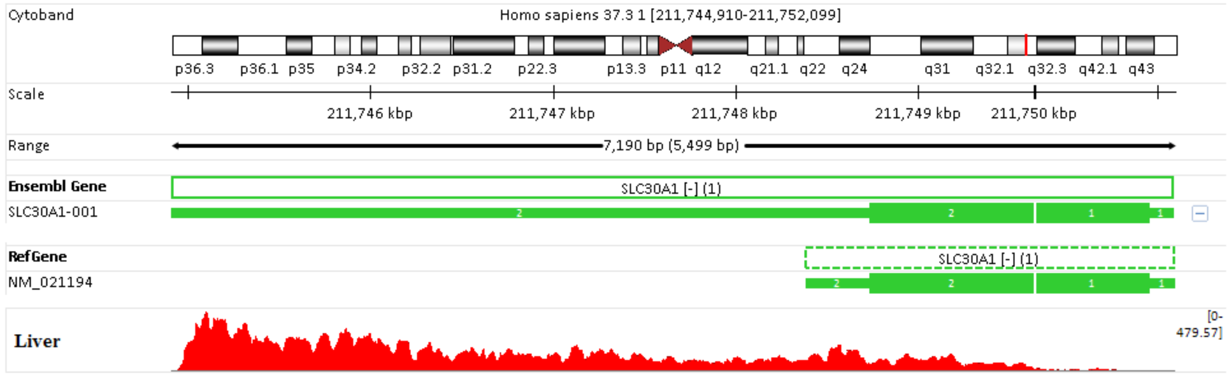

Supplementary Figure 5. The gene definitions for SLC30A1 in Ensembl and RefGene. The exons region defined in Ensembl is almost 3 times as long as in RefGene.

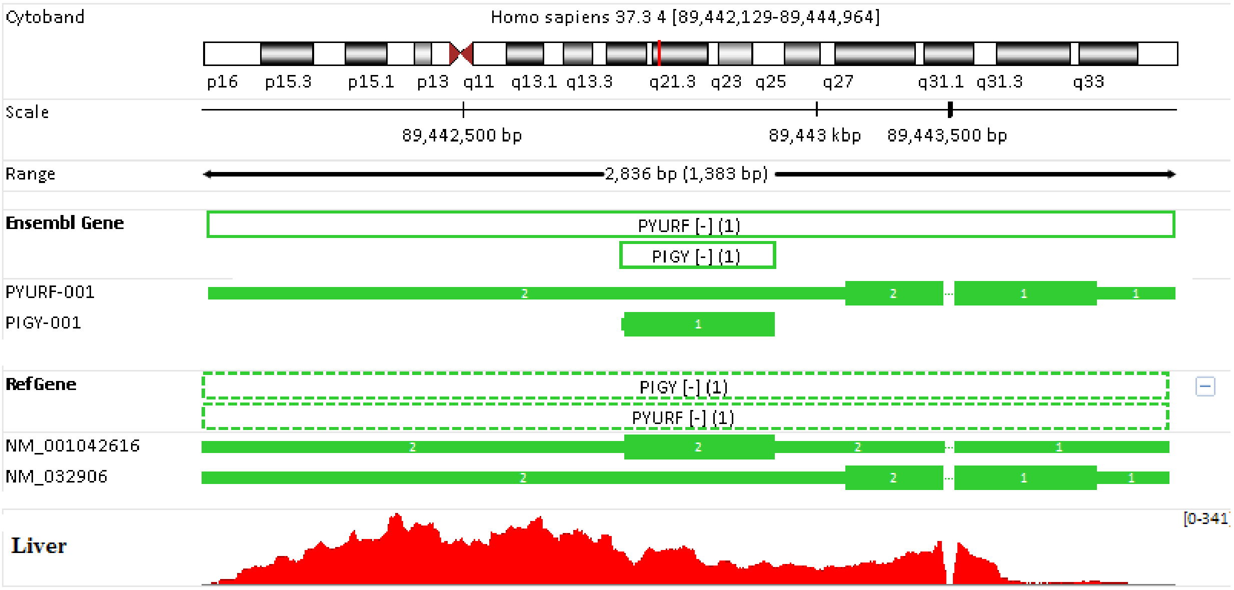

Supplementary Figure 6. The definition for PIGY in Ensembl is only 217 bp long, and is completely within gene PYURF (PIGY upstream reading frame). As a result, all reads mapped to the region of PIGY are assigned to gene PYURF only, but no read is given to PIGY. In RefGene, PIGY and PYURF encode exactly the same mRNA, though protein coding sequences are different. And thus, all reads mapped to PIGY/PYURF are equally split by these two genes.
